# Linker Molar Mass-Driven Control over Supramolecular Network Relaxation and Architecture in BTA Hydrogels

**DOI:** 10.1021/acs.macromol.5c03492

**Published:** 2026-07-10

**Authors:** Arthur Helsen, João S. Ribeiro, Ivo A. Beeren, Hans Duimel, Ruth Cardinaels, Lorenzo Moroni, Louis M. Pitet, Matthew B. Baker

**Affiliations:** † Department of Instructive Biomaterials Engineering, MERLN Institute for Technology Inspired Regenerative Medicine, Maastricht University, P.O. Box 616, 6200 MD Maastricht, The Netherlands; ‡ Advanced Functional Polymers Group, Department of Chemistry, Institute for Materials Research (IMO), Hasselt University, Martelarenlaan 42, 3500 Hasselt, Belgium; § Department of Complex Tissue Regeneration, MERLN Institute for Technology Inspired Regenerative Medicine, Maastricht University, P.O. Box 616, 6200 MD Maastricht, The Netherlands; ∥ Soft Matter, Rheology and Technology, Department of Chemical Engineering, KU Leuven, Celestijnenlaan 200J, 3001 Leuven, Belgium; ⊥ Maastricht MultiModal Molecular Imaging Institute, Maastricht University, P.O. Box 616, 6200 MD Maastricht, The Netherlands

## Abstract

The fibrous, viscoelastic extracellular matrix (ECM) directs cell fate through mechanotransduction, but recreating these time-dependent mechanics in biomaterials remains a significant challenge. Current synthetic matrices rarely reconcile fibrillar architecture, physiological stiffness, and stress relaxation, with most systems achieving only some of these hallmarks. Supramolecular benzene-1,3,5-tricarboxamide (BTA) hydrogels offer a compelling route forward, as their hydrogen-bonded nanofibers mimic ECM-like networks. Simultaneously, the reversible dynamic hydrogen bonding responsible for the assemblies enables shear thinning, self-healing, and tunable viscoelasticity. Here, three distinct BTA hydrogels were developed, distinguishable by the hydrophilic poly­(ethylene) glycol (PEG) linker length, and all hydrogelators self-assemble and form self-healing, shear thinning hydrogels. Curiously, in contrast to covalent networks, shortening the length of PEG leads to a decrease in stiffness (G′) and faster stress relaxation time scales (*t*_1/2_). Blending BTA hydrogelators with two different molar masses leads to an almost linear increase in G′ yet a more modest increase in *t*_1/2_. The hydrogels were 3D printed with good shape fidelity, and all three hydrogels are adherent, leading to a self-sustaining construct composed of three regions with distinct G′ and *t*_1/2_. These findings emphasize the power of using polymer length as an orthogonal design handle, further expanding our chemical toolbox for developing processable biomaterials with tunable viscoelasticity.

## Graphical abstract

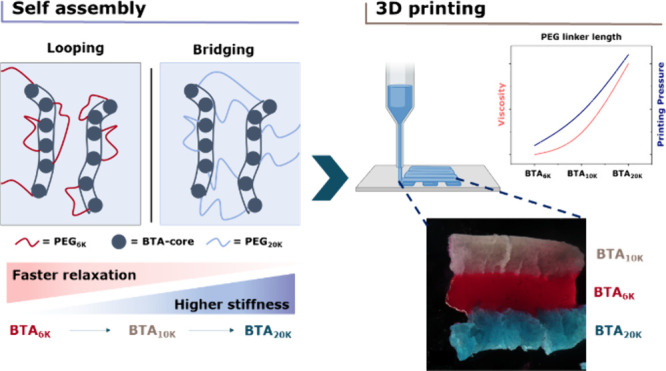

## Introduction

Recreating the dynamic mechanical and biochemical environment of native tissues using synthetic materials continues to pose a significant challenge in biomaterials design. In load-bearing tissues, the extracellular matrix (ECM) provides biochemical ligands, sequestration sites, and a fibrous, hierarchically organized microenvironment with time-dependent mechanics.^1–3^ Here, viscoelasticity, stress relaxation, and nonlinear strain stiffening govern cell spreading, migration, and lineage commitment.^4–7^ Specifically, viscoelastic relaxation on cell-relevant time scales (*t*_1/2_ ≈ 10–500 s) and fibrillar nonlinear elasticity are key regulators in mechanotransduction, integrin clustering, and nuclear mechanosensing (e.g., YAP/TAZ pathway).^8–14^

Despite significant progress within the field, synthetic matrices rarely simultaneously capture the three ECM hallmarks: fibrillar architecture, physiologically relevant stiffness, and relaxation time scales within the biologically relevant regime.^15^ Covalently cross-linked hydrogels have largely elastic properties, making them attractive as mechanically robust scaffolds.^16,17^ Nevertheless, these systems often fail to recapitulate the full dynamic complexity of heterogeneous biological systems.^18^ On the other hand, dynamic scaffolds incorporate reversible bonding capable of stress relaxation, promoting spreading/differentiation of relevant cell types, introducing more architectural complexity, and generally yield soft matrices with low moduli.^19–21^ Yet, most dynamic hydrogels lack the ECM’s nanofibrillar architecture, along with their moduli typically falling short of physiologically relevant regimes.

Supramolecular hydrogels based on benzene-1,3,5-tricarboxamide (BTA) motifs are compelling candidates to bridge this gap. Water-soluble BTAs can self-assemble via hydrogen bonding and hydrophobic interactions into one-dimensional nanofibers, resulting in cross-linked networks that resemble the native ECM architecture.^22–25^ Additionally, the reversible nature of their cross-links unlocks properties such as shear thinning, self-healing, and tunable viscoelasticity.^26^ Recently, our group has shown that substitution of the outer hydrophobic alkyl spacer can be used to modulate fiber dynamics and bulk rheology.^27^ Longer hydrophobics slowed down stress relaxation (*t*_1/2_) while maintaining near-constant stiffness, and improved 3D printing shape fidelity by increasing yield stress. In other studies, blending small-molecule BTAs with telechelic BTA-PEG-BTA polymers offers an additional design axis to tune exchange dynamics, viscoelastic properties, and cellular aggregation kinetics while maintaining a fibrillar architecture.^28,29^

In this work, we aim to explore the effect of changing the length of the PEG hydrophilic linker as an orthogonal handle to modulate the gel stiffness (G′) and relaxation times (*t*_1/2_), without compromising the crucial fibrillar assembly structure. We originally hypothesized that using shorter PEG linkers (<10 kg/mol) would lead to materials with a higher stiffness and extended stress relaxation time scales compared to longer PEG linkers (>10 kg/mol). We assumed this, given the positive correlation between cross-link density and stiffness in conventional covalent networks, and assuming that the supramolecular association would scale similarly.^30^ Surprisingly, the exact opposite correlation was found. Through self-assembly studies and oscillatory rheology, we aim to identify differences in the assembly and viscoelastic profile based on PEG linker length. Furthermore, the bulk hydrogel properties were linked with the nanoscale network architecture through small-angle X-ray scattering (SAXS) measurements. Next, by blending high- and low-molar mass BTA hydrogelators, we engineered a single material that exhibited a balance between both stiffness and fast relaxation. These trends are crucial for making molecular changes to the material when targeting specific applications. For example, the property window for chondrocytes could be G′ ∼ 10–100 kPa, with relaxation time scales *t*_1/2_ ≈ 10–200 s, as these values have been shown to promote *in vitro* osteo/chondrogenic mechanotransduction.^3^ In this system, using a short 6 kg/mol linker and increasing total polymer content to 20 wt % produced a hydrogel in this range. Next, we harnessed the material’s shear thinning and self-healing properties to 3D print scaffolds with spatial stiffness gradients and relaxation times. In this manner, our goal is to create steps toward tunable architectures that exhibit both high stiffness and fast relaxation to aid biomaterial development for load-bearing tissues.

## Experimental Section

### Materials

All chemicals were purchased from commercial sources and used as received unless stated otherwise. N,N-Diisopropylethylamine (DIPEA) solution was dried using sodium hydroxide pellets and flushed with N_2_-gas. Chloroform (CHCl_3_) was dried over molecular sieves (Sigma-Aldrich, 3A). All other solvents were >98% anhydrous. Thin-layer chromatography (TLC, precoated 0.25 mm, 60-F254 silica gel plates from Merck) was used to track reaction progress. Silica gel (40–63 μm, Sigma-Aldrich) was used to run flash column chromatography.

## Methods

### PEG-Carbonyldiimidazole Synthesis

PEG (6, 10, 20 kg/mol) was dried via azeotropic distillation using a Dean–Stark setup. Dried PEG (0.00166 mol, 1 equiv) was dissolved in 50 mL dry chloroform under vigorous stirring under Ar atmosphere. Then, 1,1′-carbonyldiimidazole (11.6 equiv, 0.0136 mol) was dissolved in 10 mL dry chloroform and added dropwise to the PEG solution while under Ar atmosphere. The reaction mixture was stirred for 20h at 37 °C while maintaining inert atmosphere. The mixture was precipitated in ice-cold diethyl ether twice and dried overnight on a high vacuum setup. The products PEGCDI_6K_, PEGCDI_10K_, and PEGCDI_20K_ were isolated as a white solid with >80% yield. ^1^H NMR (400 MHz, CDCl_3_): δ 8.15 (s, 2H, Ar), 7.43 (s, 2H, Ar), 7.06 (s, 2H, Ar), 4.55 (t, 4H, CH2OC=O), 3.8–3.4 (b, 571H (PEG_6K_), 952H (PEG_10K_), 1904H (PEG_20K_) O–CH_2_CH_2_–O).

### PEG-Diaminododecane Synthesis

1,12-Diaminododecane (16 equiv, 4.51 mmol) was weighed and dissolved in 50 mL anhydrous DMF in a 250 mL round-bottom flask. The solution was heated to 70 °C under an Ar atmosphere. Next, PEG-CDI (1 equiv, 0.282 mmol) was dissolved in 90 mL anhydrous DMF and added dropwise to the solution while stirring under Ar atmosphere. The reaction was left running for 48h at 70 °C. The reaction mixture was precipitated three times in excess ice-cold diethyl ether and put on high vacuum overnight to dry. The products PEGDAD_6K_, PEGDAD_10K_, and PEGDAD_20K_ were isolated with >80% yield as a white solid. ^1^H NMR (400 MHz, CDCl_3_): δ 4.83 (s, 2H, NHC=O), 4.20 (t, 4H, CH_2_OC=O), 3.8–3.4 (b, 571H (PEG_6K_), 952H (PEG_10K_), 1904H (PEG_20K_) O–CH_2_CH_2_–O), 3.15 (q, 4H, CH_2_NHC=O), 2.68 (t, 4H, CH_2_NH_2_), 1.47–1.25 (m, 40H, aliphatic).

### BTE-F_5_Ph Synthesis

F_5_Ph (15.57 g, 0.0846 mol, 4.5 equiv) was dissolved in 40 mL dry DCM in a 250 mL round-bottom flask under Ar atmosphere. An ice bath was used to cool down the reaction mixture for the next steps. Dry diisopropylethylamine (DIPEA, 9.72 g, 0.0752 mol, 4 equiv) was dissolved in 30 mL dry DCM and added dropwise to the F_5_Ph-solution. BTCl (5 g, 0.0188 mol, 1 equiv) was dissolved in 20 mL dry DCM and added dropwise to the reaction mixture while stirring vigorously. The ice bath was removed after 20 min. The reaction was left stirring for 2 h, with TLC samples taken after 40 min and 2 h, respectively. The reaction mixture was filtered through filter paper before isolating the trisubstituted product over silica (100% DCM). The successful isolation was monitored via TLC. The final product was dried via rotary evaporation and left under high vacuum overnight. It was obtained as a white solid (83% yield). ^1^H NMR (400 MHz, CDCl_3_) δ 9.29 (s, 3H). ^13^C NMR (100 MHz, CDCl_3_) 160.5, 142.5, 141.3, 139.9, 139.3, 138.8, 137.7, 136.8, 129.4, 124.7

### BDA Synthesis (C_12_C_12_)

In an air-free, dry three-neck 250 mL round-bottom flask, BTE-F_5_Ph (4 g, 5.64 mmol, 1 equiv) was added and dissolved in 20 mL dry DCM while stirring in Ar atmosphere. The RBF was placed in an ice bath to cool the reaction mixture. Dry DIPEA (1.53 g, 11.844 mmol, 2.1 equiv) was dissolved in 10 mL dry DCM and added dropwise to the solution while stirring. Dodecylamine (2.09 g, 11.28 mmol, 2 equiv) was dissolved in 20 mL anhydrous DCM and added dropwise to the mixture over 10 min while stirring and under Ar-flow. The ice bath was removed after 20 min. The reaction mixture was stirred for 2 h and monitored via TLC at 40 min and 2 h. The solvent was evaporated under reduced pressure and dried under high vacuum overnight. The product was redissolved in a minimal amount of DCM and isolated via silica flash column chromatography using acetonitrile/DCM (5/95) as eluent. The isolation was monitored using TLC. The product was obtained as a white powder with 54% yield (3.2 g). ^1^H NMR (400 MHz, CDCl_3_) δ: 8.66 (2H, d), 8.50 (1H, t), 6.39 (2H, t), 3.48 (4H, q), 1.61 (4H, m), 1.4–1.2 (36H, m), 0.86 (6H, t) ^13^C NMR (100 MHz, CDCl_3_) δ: 165.1, 161.5, 136.1, 131.3, 127.8, 40.5, 31.9, 29.3, 26.9, 22.7, 14.8

### BTA_6K_ Synthesis

In a dry, three-neck 250 mL RBF, BDA_C12C12_ (250 mg, 0.719 mmol, 2.3 equiv) was dissolved in 20 mL anhydrous DCM while under argon. DIPEA (120 mg, 0.93 mmol, 3.1 equiv) was dissolved in 10 mL dry DCM and added dropwise while stirring. PEG-DAD_6K_ (1 g, 0.3 mmol, 1 equiv) was dissolved in 15 mL anhydrous DCM and added dropwise to the reaction mixture while stirring under argon. The reaction was left to run for 48 h at room temperature while under inert atmosphere. The mixture was precipitated twice in ice-cold diethyl ether to obtain a white powder and concentrated *in vacuo* overnight. The product was dissolved in methanol and dialyzed against methanol for 3 days. Methanol was removed under reduced pressure to obtain the product as a white powder with 49% yield (0.6 g). ^1^H NMR (400 MHz, DMSO-*d*_6_) δ: 8.62 (t, 6H, NH­(C=O)), 8.35 (s, 6H, Ar), 7.16 (t, 2H, CH_2_NH­(C=O)­O), 4.03 (t, 4H, NH­(C=O)­OCH_2_), 3.6–3.37 (bs, 571H, O-(CH_2_)_2_–O), 3.26 (t, 12H, (C=O)­NHCH^2^), 2.92 (q, 4H, CH^2^NH­(C=O)­O), 1.59–1.15 (mm, 120H, aliphatic), 0.84 (dt, 12H, CH_2_CH_3_, aliphatic).

### BTA_10K_ Synthesis

In a dry, three-neck 250 mL RBF, BDA_C12C12_ (170 mg, 0.24 mmol, 2.5 equiv) was dissolved in 10 mL anhydrous DCM while under argon. DIPEA (37.1 mg, 0.288 mmol, 3 equiv) was dissolved in 10 mL dry DCM and added dropwise to the solution. PEG-DAD_10K_ (1 g, 0.096 mmol, 1 equiv) was dissolved in 15 mL anhydrous DCM and added dropwise to the solution while stirring under Ar-flow. The reaction was left to run for 48 h at room temperature while under inert atmosphere. The mixture was precipitated twice in ice-cold diethyl ether to obtain a white powder and concentrated *in vacuo* overnight. The product was dissolved in methanol and dialyzed against methanol for 3 days. Methanol was removed under reduced pressure to obtain the product as a white powder with 53% yield (0.6 g). ^1^H NMR (400 MHz, DMSO-*d*_6_) δ: 8.62 (t, 6H, NH­(C=O)), 8.35 (s, 6H, Ar), 7.16 (t, 2H, CH_2_NH­(C=O)­O), 4.02 (t, 4H, NH­(C=O)­OCH_2_), 3.6–3.37 (bs, 952H, O-(CH_2_)_2_–O), 3.26 (t, 12H, (C=O)­NHCH_2_), 2.92 (q, 4H, CH_2_NH­(C=O)­O), 1.59–1.15 (mm, 120H, aliphatic), 0.84 (dt, 12H, CH_2_CH_3_, aliphatic).

### BTA_20K_ Synthesis

In a dry, three-neck 250 mL RBF, BDA_C12C12_ (98.8 mg, 0.14 mmol, 2.2 equiv) was dissolved in 10 mL anhydrous DCM while under argon. DIPEA (28 mg, 0.226 mmol, 3 equiv) was dissolved in 10 mL dry DCM and added dropwise to the solution. PEG-DAD_20K_ (1.20 g, 0.06 mmol, 0.92 equiv) was dissolved in 15 mL anhydrous DCM and added dropwise to the solution while stirring under Ar-flow. The reaction was left to run for 48 h at room temperature while under inert atmosphere. The mixture was precipitated twice in ice-cold diethyl ether to obtain a white powder and concentrated *in vacuo* overnight. The product was dissolved in methanol and dialyzed against methanol for 3 days. Methanol was removed under reduced pressure to obtain the product as a white powder with 52% yield (1.13 g). ^1^H NMR (400 MHz, DMSO-*d*_6_) δ: 8.62 (t, 6H, NH­(C=O)), 8.35 (s, 6H, Ar), 7.16 (t, 2H, CH_2_NH­(C=O)­O), 4.03 (t, 4H, NH­(C=O)­OCH_2_), 3.6–3.37 (bs, 1904H, O-(CH_2_)_2_–O), 3.26 (t, 12H, (C=O)­NHCH_2_), 2.92 (q, 4H, CH_2_NH­(C=O)­O), 1.59–1.15 (mm, 120H, aliphatic), 0.84 (dt, 12H, CH_2_CH_3_, aliphatic).

### Nuclear Magnetic Resonance (NMR) Analysis and Sample Preparation

NMR measurements were performed using a JEOL instrument (400 MHz) at 128 scans. Samples (10–15 mg/mL) were dissolved in CDCl_3_ unless specified otherwise for ^1^H NMR and ^13^C NMR. Analysis was performed using MestReNova. Chemical shift values are provided in ppm (δ) relative to the residual solvent peak. Splitting patterns were labeled: s, singlet; bs, broad singlet; d, doublet; dd, double doublet; t, triplet; q, quartet; p, pentet; m, multiplet; b, broad.

### Size Exclusion Chromatography (SEC) Measurements

SEC measurements were performed using a TOSOH EcoSEC instrument (CHCl_3_). Samples were prepared at 5 mg/mL in CHCl_3_ with 100 ppm toluene internal standard and filtered using a 0.2 μM Teflon filter. Calibrations were performed using polystyrene internal standards. Raw SEC data was processed and analyzed using OriginPro 2020.

### MALDI-ToF Spectrometry

Matrix-assisted laser desorption/ionization - time-of-flight (MALDI–ToF) mass spectra were recorded on a Bruker Daltonics ultrafleXtreme MALDI/ToF-ToF system. α-Cyano-4-hydroxycinnamic acid in 50% water/50% acetonitrile containing 0.1% TFA was used as a matrix.1 μL of this solution was spotted onto an MTP Anchorchip 600/384 MALDI plate. Analysis was performed using MestReNova.

### Nile Red Studies

For all three macromolecules (BTA_6K,10K,20K_), samples were prepared with a concentration of 1, 2, 5, and 10 mg/mL. Solid polymer was weighed and dissolved in 50 μL methanol (dispersed). 950 μL demineralized water was added to the sample and vortexed, before performing three heating–cooling cycles. The solutions were heated for 1 min using an aluminum heating block (80 °C), vortexed for 15 s and cooled down to room temperature on the benchtop. Samples were aged overnight (4 °C) and measured the next day. A 471 μM Nile Red stock solution was prepared in DMSO, and 10.62 μL stock solution was added to the samples to obtain a final concentration of 5 μM. Samples were stored in the dark for 30 min before measurement at room temperature. Emission wavelength spectra were recorded at λ_exc_ = 540 nm using an Agilent Cary Eclipse fluorescence spectrophotometer.

### Cryo-TEM

Samples were prepared at either 5 or 10 mg/mL. Polymers were dissolved in 50 μL methanol (dispersed), after which 950 μL demi-water was added. The sample solution was heat–cooled as described in the ‘Nile Red studies’ section. Samples were stored overnight (4 °C) before imaging.

Sample vitrification was performed using the FEI Vitrobot Mark IV automated vitrification robot. R2/2 Quantifoil Jena grids were used for Cryo-TEM and purchased from Quantifoil Micro Tools GmbH. Before the vitrification procedure (3 μL aliquots, blotting time 3–4s, −5 mm blotting offset, 100% relative humidity), the grids were surface glow discharged using a Cressington 208 carbon coater operating at 7 mA for 30 s. Cryo-TEM experiments were performed on a FEI Arctica 200 kV microscope equipped with a FEG operating at 200 kV. Images were recorded using a Falcon III camera and analyzed using Fiji ImageJ software.

### Dynamic Light Scattering (DLS)

Dynamic Light Scattering measurements were performed using a Malvern Zetasizer Nano (Sysmex, NL) at room temperature to obtain the apparent hydrodynamic diameter (Z-average) and intensity-weighted size distribution of the BTA hydrogelators at 10 mg/mL. The sample preparation follows the protocol described in the ‘Nile Red Studies’ methods section.

### Hydrogel Formation

Solid polymers were weighed in a glass vial and dissolved in deionized water. Water volume was adjusted according to the specified concentration, ranging from 3.33–20 wt %. The glass vial was heated using an aluminum heating block (80 °C), vortexed, and cooled to room temperature. This cycle was repeated three times. The resulting gel was allowed to cool down to room temperature and stored at 4 °C overnight before measuring the next day.

### CGC Experiments

Hydrogels were prepared according to the ‘Hydrogel Formation’ section at 10 wt %. Gels were diluted in series, as described in the results section, and vial inversion was performed after 24 h. The vial was inverted, after which the flow was observed for 30 s. If there was no flow within 30 s, it is qualitatively described as a gel. If flow was observed within this time frame, it is not considered a gel.

### Macroscopic Self-Healing

Hydrogels were prepared according to the ‘Hydrogel formation’ section and pressed into circular molds using a spatula. Two drops of green, food-grade colorant were added to the hydrogel to facilitate visual differentiation. Afterward, they were cut into two pieces using a surgical blade and pushed back together using a spatula and some force. This leads to a macroscopically uniform material that is self-supporting. Multiple halves of the hydrogel were united to obtain a ladder-like pattern, which was self-sustaining while hanging from a spatula.

### Injectability

After forming the hydrogels according to the ‘Hydrogel formation’ section at 10 wt %, the hydrogels were removed from the vials and transferred into a 1 mL syringe. A 21-gauge needle was attached, and the materials were extruded by hand onto a glass cover plate for visualization.

### Viscoelastic Behavior

Rheological measurements were performed using an Anton Paar MCR 702 instrument with a sandblasted Peltier plate at 25 °C and a 25 mm cone–plate geometry (2° angle). First, a strain sweep on 10 wt % hydrogels determined the linear viscoelastic regime (LVR). 1% For the following rheological measurements, 1% strain was chosen as it is within the LVR of all hydrogels. An amplitude sweep was performed from 0.1–400% strain at 1 rad/s to remove any mechanical history within the gels. Next, a time sweep (1% strain, 1 rad/s) was performed to allow sample equilibration and healing. This was followed by a stress relaxation measurement (1% strain) for up to 5000 s. Frequency sweeps were performed from 100 rad/s to 0.001 rad/s at 1% strain. A second frequency sweep was conducted immediately after the first one to confirm reproducibility and exclude sample drying effects. No significant sample drying (i.e., no increase in G′) was observed when using a solvent trap containing wet cotton swabs. Self-healing tests were carried out at 1 rad/s using a large amplitude oscillatory shear (LAOS) step protocol. A time sweep was performed at 1% strain for 120 s to monitor the baseline modulus, followed by a rapid strain step to 400% that was maintained for 200 s to induce network breakdown. The strain was then rapidly reduced back to 1% and held for 100 s to monitor recovery. This sequence was repeated four times to assess the reproducibility of recovery after large deformation. For viscosity measurements, a flow sweep was performed on a DHR-20 (TA Instruments) from 10^–3^ to 10^3^ 1/s at 25 °C. Steady-state sensing was enabled in TRIOS software with an equilibration time of 45 s and a sample time of 15 s, respectively. Data points were acquired with a tolerance of 5% between three consecutive points. For frequency sweeps and stress relaxation measurements, reproducibility was assessed in a different lab using a DHR-20 (TA Instruments) at 25 °C using a cone plate (20 mm, 2° angle). Data and statistical analysis (mean and SD, and one-way ANOVA on log-transformed data with Tukey posthoc) were performed using OriginPro 2020 (Academic) software.

### Small-Angle X-ray Scattering (SAXS) Measurements

SAXS (1.033 A wavelength, 12 keV) analysis was performed on the BM26 (DUBBLE) Beamline at the European Synchrotron Radiation Facility (ESRF) in Grenoble, France. Samples were weighed and loaded into standard DSC pans and lids (4–5 mg per sample). SAXS was performed using a 1.4 m camera length at temperature-controlled conditions of 20 and 37 °C, with a 5 min stabilization time at each temperature. Data was collected and processed in Igor.

### 3D Printing

BTA hydrogels were printed at a concentration of 10 wt %. An extrusion-based CellInk BioX bioprinter was used to investigate printability. Printing was performed at room temperature. 20-gauge (ID = 0.81 mm) needles were fixed to a 3 mL syringe for extrusion. The extrusion pressure was varied from 70–200 kPa depending on the BTA hydrogel formulation. The printing speed was kept at 0.5 mm/s for all formulations. Infill density was set to 100%, and the distance from the needle to the surface was optimized to ensure that the leading edge of the flow aligned with the needle. All hydrogels were extruded in a continuous filament line. Two structures were printed in a grid-like structure (two layers, 10 × 10 grid, 15 mm width and length). All printing was performed on a 48-well plate or a plastic Petri dish. Structures were imaged using a Nikon SMZ25 stereomicroscope, and images were analyzed using NIS Elements D5.02 software. Analysis was performed using OriginPro 2020 (Academic) software.

## Results and Discussion

### BTA Macromolecule Synthesis

To systematically investigate the effect of hydrophilic domain length on the supramolecular properties, we synthesized BTA derivatives with varying PEG linker length, utilizing three precursors with molar mass of 6, 10, and 20 kg/mol (Scheme 1). In this manuscript, all synthesized macromolecules will be referred to by the PEG molar mass used during synthesis in subscript, e.g., BTA_6K_. PEG-CDI (**1**_**a, b, c**_) coupling was successful for all three PEG molar masses, with minimal variation in functionalization % across the series, as determined by ^1^H NMR analysis (Figures S1–S3). Subsequently, PEG-CDI was end-functionalized with 1,12-diaminododecane (DAD) in DMF to obtain PEG-DAD (**2**_**a, b, c**_) for all molar masses (Figures S4–S6). Reaction conditions were maintained consistent for each PEG molar mass and controlled through carefully adjusting DAD equivalents. As reported by Hafeez et al.^31^ and Rijns et al.,^32^ the chain extension observed for PEG_20K_-DAD and PEG_10K_-DAD was also found in the PEG_6K_-DAD polymers, as confirmed by GPC of the final product (Figure S7).

**Scheme 1:**
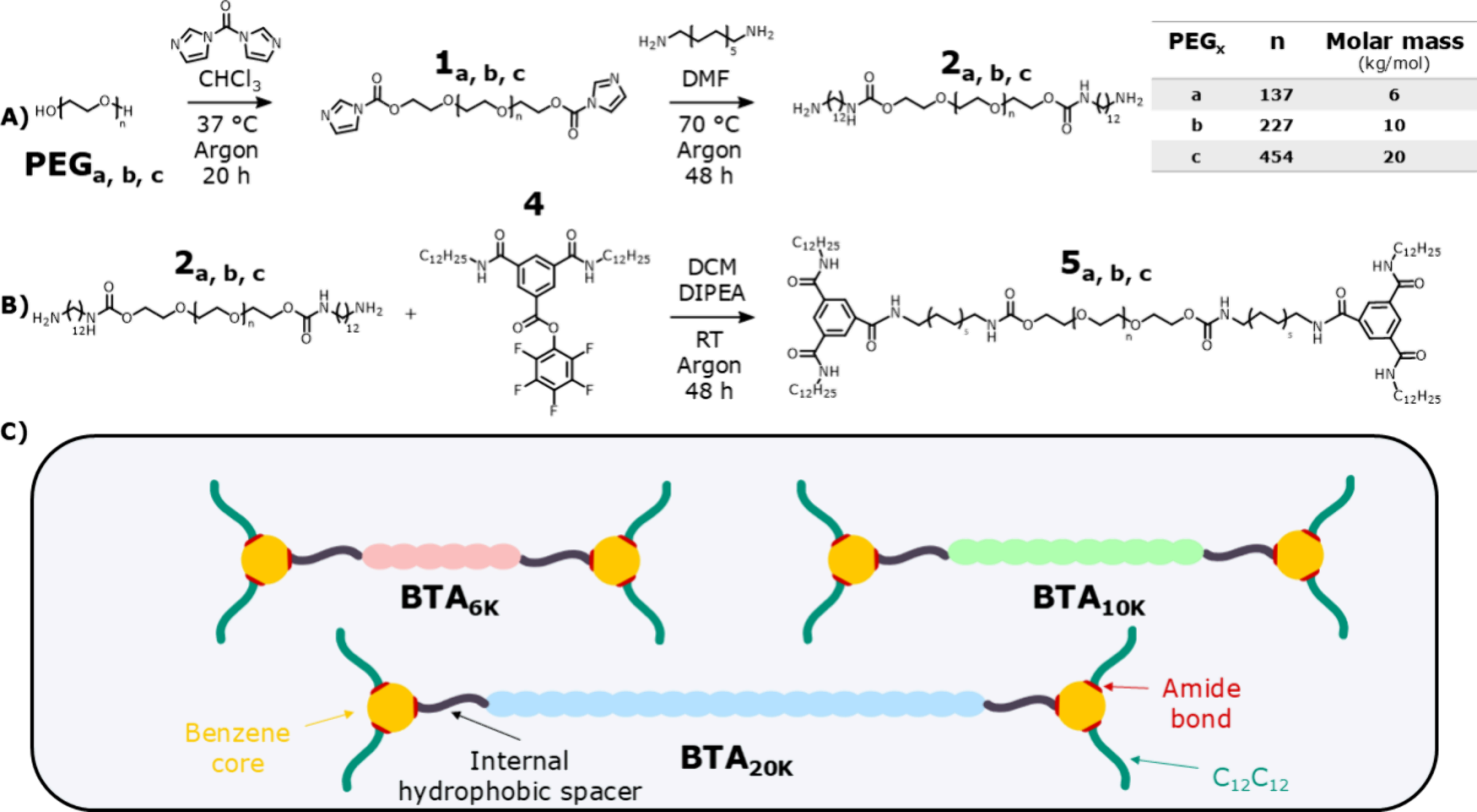
Synthesis Procedure of BTA Hydrogelators^a^

a A) Synthetic pathway where **2** (PEG-diaminododecane) is synthesized using PEG with variable molar mass (a, b, c = 6, 10, 20 kg/mol, respectively) starting from **1** (PEG-CDI). B) **4** (BDA) was synthesized with 2 × C_12_ hydrophobic linkers and coupled to **2** yielding **5** (BTA macromolecules). C) Schematic representation of BTA hydrogelators with variable PEG linker length.

Having successfully synthesized the hydrophilic linker, the next step was to develop the BDA units. To ensure consistency with prior work, the outer and inner hydrophobic spacers were kept at 12 methylene units.^31^ In short, the symmetrical BTE-F_5_Ph was used to couple the aliphatic dodecylamine, isolating the disubstituted, desymmetrized BDA_C12_ derivatives (Scheme S1). The successful synthesis and isolation of BTE-F_5_Ph (**3**) and BDA_C12_ (**4**) was confirmed by ^1^H- and ^13^C NMR spectroscopy (Figures S8–11). Yield (>50%) was in line with our previously published protocol.^31^

The final BTA macromolecules (**5**_**a, b, c**_) – BTA_6K_, BTA_10K_, and BTA_20K_ – were obtained by coupling BDA_C12_ to their respective PEG-DAD linkers. Due to the robustness of the desymmetrization reaction, all three BTA macromolecules were successfully formed and isolated as pure products with yields (>50%), as supported by ^1^H NMR, GPC, and MALDI-TOF (Table S1–S2; Figures S7, S12–S14).

### Self-Assembly Studies in Dilute Conditions

Having developed three distinct BTA macromolecules with different molar masses, the first step was to investigate their self-assembly behavior in aqueous solutions. Our previous work showed that the BTA_20K_ hydrogelators tend to self-assemble via supramolecular interactions into fibers in aqueous solution.^27,31^ Hence, the same hypothesis was tested for the BTA_10K_ and BTA_6K_ formulations. To probe this, Nile Red was used as a lipophilic, solvatochromic dye, which localizes inside the hydrophobic pocket formed by the aliphatic side chains.^33^ Comparing the differences in maximum emission wavelength (λ_em,max_) of Nile Red across the series allowed us to determine if PEG hydrophilic linker length impacts the polarity of the environment, and by extension, the supramolecular assembly (Figure 1). Sample preparation is described in the ‘Nile Red Studies’ method section. In Figure 1A, the emission wavelength (λ_em_) of Nile Red is given for all BTA hydrogelators at various concentrations. At the same BTA concentration (50 μM), the maximum emission wavelength of Nile Red remains relatively consistent across the series (Figure S15). A trend emerges when comparing the maximum emission wavelength (λ_em,max_) across the series, which blueshifts (i.e., a decrease in polarity) with increasing polymer concentration (Figure S16). This concentration dependency is largely consistent across the BTA series, indicating that the polarity of the supramolecular assemblies is likely independent of the hydrophilic linker length. Second, this shift in λ_em,max_ suggests that more water is expelled from the assemblies, leading to a more apolar environment at higher concentrations. The emission spectra remained constant over 7 days, showing no significant changes in polarity and suggesting these assemblies remain stable on this time scale (Figure S17).

**Figure 1:**
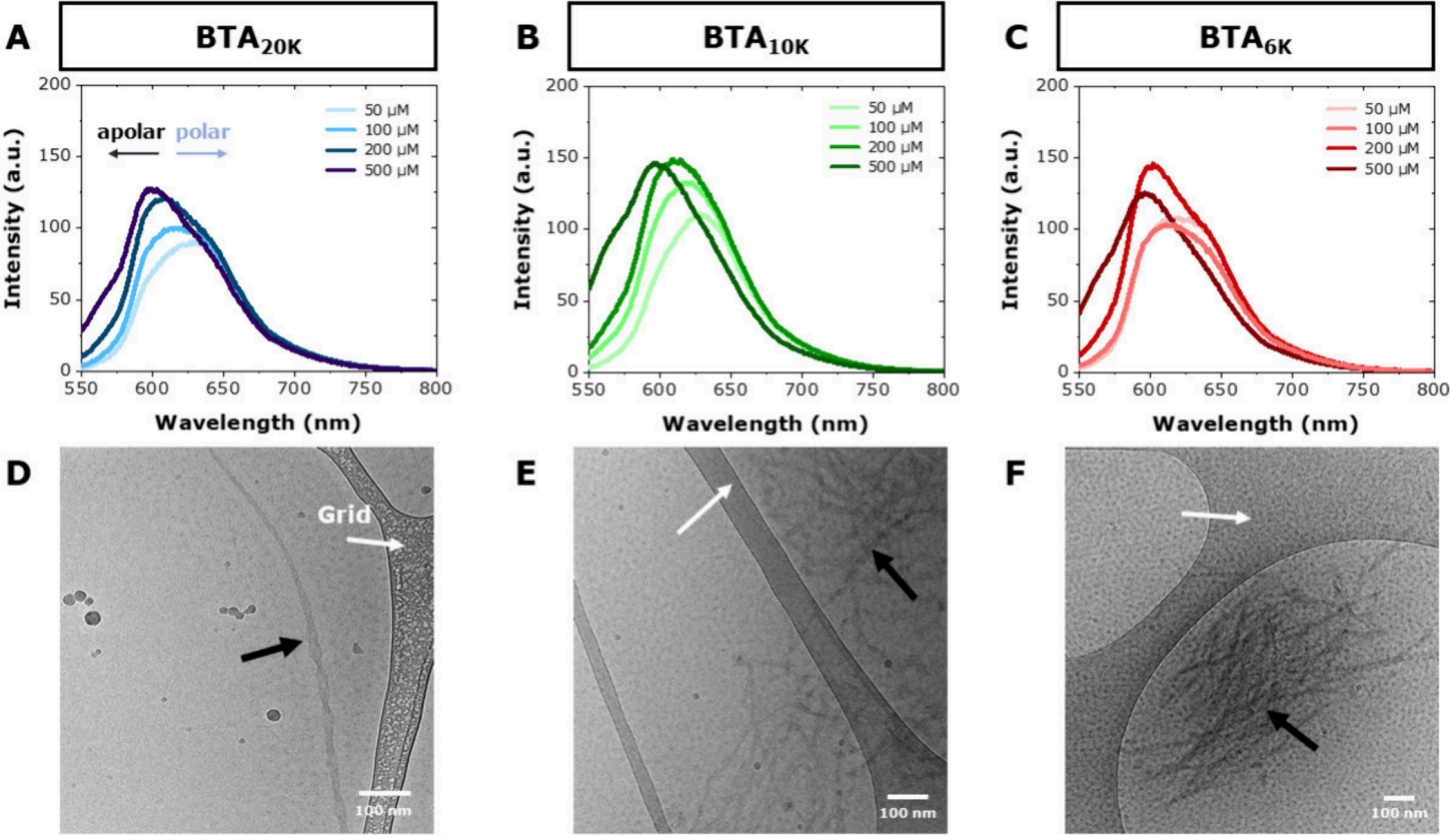
**Characterization of BTA macromolecules in dilute solution**. A-C) fluorescence emission intensity of Nile Red in BTA_6K,10k&20K_. λ_em,max_ is similar for all BTA formulations tested at the same concentrations. An increase in BTA concentration leads to more hydrophobic assemblies (∼29 nm shift) for all BTAs. D-F) Cryo-TEM images of BTA formulations (BTA_20K_ = 5 mg/mL; BTA_6&10K_ = 10 mg/mL) showing fibrous self-assemblies (black arrows) on a micrometer scale. More bundling is present when PEG *M*_w_ is decreased. Solvent = H_2_O:MeOH (95:5).

Although the λ_emission,max_ shifts with varying concentration within each material, it remains constant across all BTAs at a given concentration. This suggests that the three BTA hydrogelators likely self-assemble into comparable supramolecular architectures in dilute solution. To probe this, a more direct approach was taken to identify the formed morphologies using cryogenic transmission electron microscopy (cryo-TEM). BTA hydrogelators were diluted to 10 mg/mL (BTA_6K_ & BTA_10K_) and 5 mg/mL (BTA_20K_). The concentration of BTA_20K_ was halved due to complete saturation of the cryo-TEM grid at 10 mg/mL, making it impossible to identify structures. Cryo-TEM images are shown in Figure 1D–F for all three BTAs. All three hydrogelators appear to self-assemble into similar morphologies, mainly corresponding to a fiber-like architecture consistent with previous reports.^31,32,34–36^ These fibrillar morphologies show a flat, crossed-over appearance with an individual diameter of 17.4 ± 3 nm, 18.6 ± 4 nm, and 20.4 ± 5 nm for BTA_6K_, BTA_10K_, and BTA_20K_, respectively (Figure S18). This corroborates the Nile Red assay findings, strengthening the observation that all three BTA hydrogelators self-assemble into fibrillar morphologies in aqueous solution, independent of PEG molar mass.

Nile Red spectra and Cryo-TEM analysis indicate that all three BTA hydrogelators self-assemble into comparable fibrous architectures. The modest increase in fiber diameter observed with longer PEG linkers suggests that linker length can modulate BTA packing or fiber bundling. We believe this effect can be attributed to how PEG linkers connect adjacent BTA stacks: shorter linkers are more prone to looping and form shorter bridges, altering the local hydration of BTA stacks. As a result, assemblies with shorter PEG chains could undergo stronger hydrophobic collapse than those with longer PEG chains. Directly resolving loop vs bridge topologies is not feasible from our cryo-TEM data, as the PEG linkers are not directly visible, and because the 2D projection of a 3D vitrified network prevents unambiguous assignment due to overlap of the beam path. An attempt was made to understand the structures by using Dynamic Light Scattering (DLS) to assess differences in the populations of self-assembled BTA_20K_ and BTA_6K_ in dilute solution (Figure S19). Although a broader, intensity-weighted distribution was obtained for the BTA_6K_ hydrogelator compared to BTA_20K_, no clear trends emerged to further resolve looping vs bridging. A more rigorous topological assessment would require, e.g., 3D cryo-electron tomography and/or contrast-selective scattering approaches. However, such molecular-level details are experimentally difficult to assess, leaving this area open for future exploration.

The replication of fibrillar morphologies is key to mimicking the architecture and mechanics of the native ECM. The synthetic fibrils described above, with diameters of around 20 nm, approach the scale of collagen II fibrils found in cartilage tissue, which have diameters ranging from 20 to 60 nm, depending on age and mechanical load.^37^ As such, these supramolecular systems are considered promising candidates to develop hydrogels with enhanced mechanical properties and biological relevance for biomaterial development.

### Hydrogel Macroscopic Behavior

#### Critical Gelation Concentration

After investigating their behavior in dilute solutions, BTA hydrogelators were subjected to a qualitative vial inversion test. This simple assay was included as an intuitive, comparative demonstration of self-supporting behavior across PEG linker lengths, rather than a quantitative determination of the gel point. All three BTA hydrogelators were found to form self-supporting hydrogels at 7.5 wt % (Figure 2A), and complementary oscillatory rheology confirms gel-like behavior at 10 wt %, with tan δ < 1 (G′ > G′′) for all materials above ∼0.01 rad/s (Figure S20). To qualitatively estimate an apparent critical gelation concentration (CGC) by vial inversion, a 10 wt % hydrogel was serially diluted, equilibrated overnight, and assessed by observing flow for 30 s after inversion. Using this method, the apparent CGC was found to be ≈6.0 wt %, 3.5 wt % and 1.9 wt % for BTA_6K_, BTA_10K_, and BTA_20K_, respectively. A trend is observed that the apparent CGC is increased for BTAs with lower PEG molar mass, and scales almost linearly with the difference in PEG length. Decreased physical interactions, such as polymer entanglements and looping rather than bridging of shorter PEG chains, can rationalize this result. This difference in apparent CGC confirms our suspicion that, in addition to the hydrophobic aliphatic side chains, the hydrophilic linker can be tuned to achieve materials with distinct hydrogel behavior.

**Figure 2:**
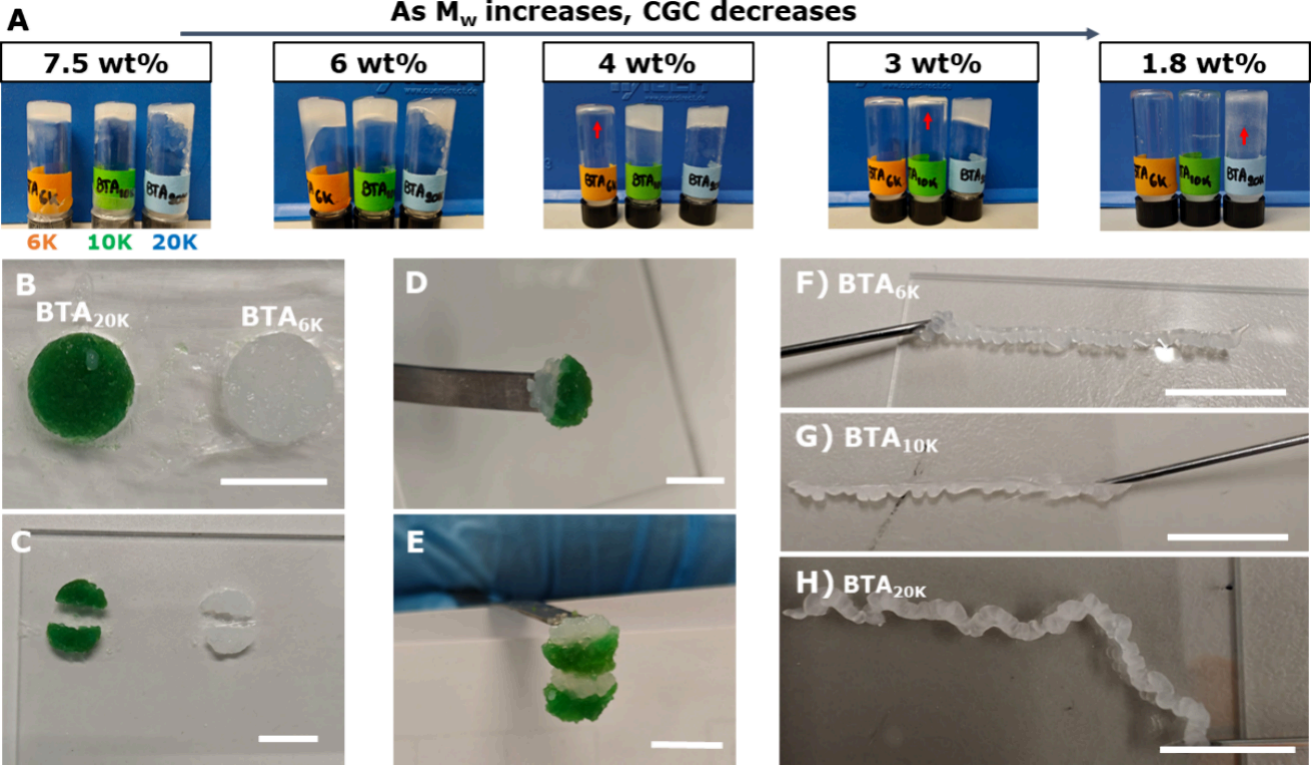
**Hydrogel formation and macroscopic properties**. A) All BTA formulations form hydrogels at 7.5 wt % according to the vial inversion method. The apparent critical gelation concentration (CGC) depends on PEG *M*_w_, with an inverse relation between *M*_w_ and apparent CGC. B) BTA_20K_ (green) and BTA_6K_ (white) can be fit into a mold of interest. C) Hydrogels can be cut with a spatula using relatively little force. D) Two individual pieces of BTA_20K_ and BTA_6K_ were united by pushing them together, forming a uniform, self-sustaining hydrogel. E) Multiple pieces adhere together in a ladder-like pattern. F) BTA_6K_, G) BTA_10K_, and H) BTA_20K_ can be injected through a 21-gauge needle into fibers and larger self-sustaining structures. Scale bar corresponds to 1 cm.

#### Moldability, Self-Healing, and Injection

As shown in Figure 2B, BTA hydrogels can be transferred into a defined shape using a spatula, demonstrating their cohesive and moldable character. Upon demolding, the gels were cut with a scalpel, where higher molar mass formulations resisted cutting more strongly than the lower molar mass BTAs (Figure 2C). When both BTA_20K_ and BTA_6K_ hydrogel segments were brought into contact, they fused into a two-component construct that retained its integrity when lifted with a spatula (Figure 2D). This self-supporting behavior was maintained even when four distinct hydrogel pieces were combined into a ladder-like arrangement (Figure 2E), highlighting the ability to form composite materials without visible interfacial separation. This result illustrates the potential of mixing BTAs with different linker lengths to create hydrogel constructs with mechanical gradients. All formulations displayed shear thinning behavior, and injection through a 21-gauge needle was followed by rapid recovery to their initial state (Figures 2F–H). The extrusion force increased significantly with PEG molar mass, where BTA_6K_ required less effort than BTA_10K_ and BTA_20K_. This combination of moldability, injectability, and capacity to form composite materials showcases the versatility of BTA hydrogels for applications in biofabrication.

### Tuning Viscoelasticity and Stress Relaxation

#### PEG Molar Mass Drives Viscoelasticity

The vial inversion test revealed significant differences in the flow behavior of the hydrogels, with flow increasing as the PEG molar mass decreased. To quantitatively corroborate these findings, oscillatory shear rheology was used. The respective storage (G′) and loss (G′′) moduli were determined and compared across the series. Based on the macroscopic behavior, we expect that G′ depends on PEG linker length. In doing so, we aim to develop a library of materials with unique viscoelastic properties.

Through an amplitude sweep performed at 1 rad/s, 1% strain was found to be within the linear viscoelastic regime (LVR) for all three materials. The yield strain of 10 wt % hydrogels was found to remain relatively constant with increasing PEG linker length (Figure 3A). The stiffness (G′), on the other hand, was found to increase from 2.4 kPa (BTA_6K_) to 7.8 kPa (BTA_10K_) and 12.4 kPa (BTA_20K_). Repeat measurements show good agreement in G′ across independent batches and two rheometers for each 10 wt % formulation (Figure S21A, B). The G′ for hydrogels at 15 and 20 wt % increased significantly as compared to those at 10 wt % (Figure S22A, B). Rheological measurements for BTA_20K_ at 15 and 20 wt % were not performed, as the high normal forces after sample loading required long relaxation times during which the samples dry out, preventing reliable analysis. These findings are in agreement with the vial inversion tests but a stark contrast to our initial hypothesis, where we assumed that BTA_6K_ would have a higher G′ compared to BTA_20K_. We were surprised by this result, as common covalently cross-linked networks typically exhibit the opposite correlation between modulus and molecular weight between cross-links.^30^

**Figure 3:**
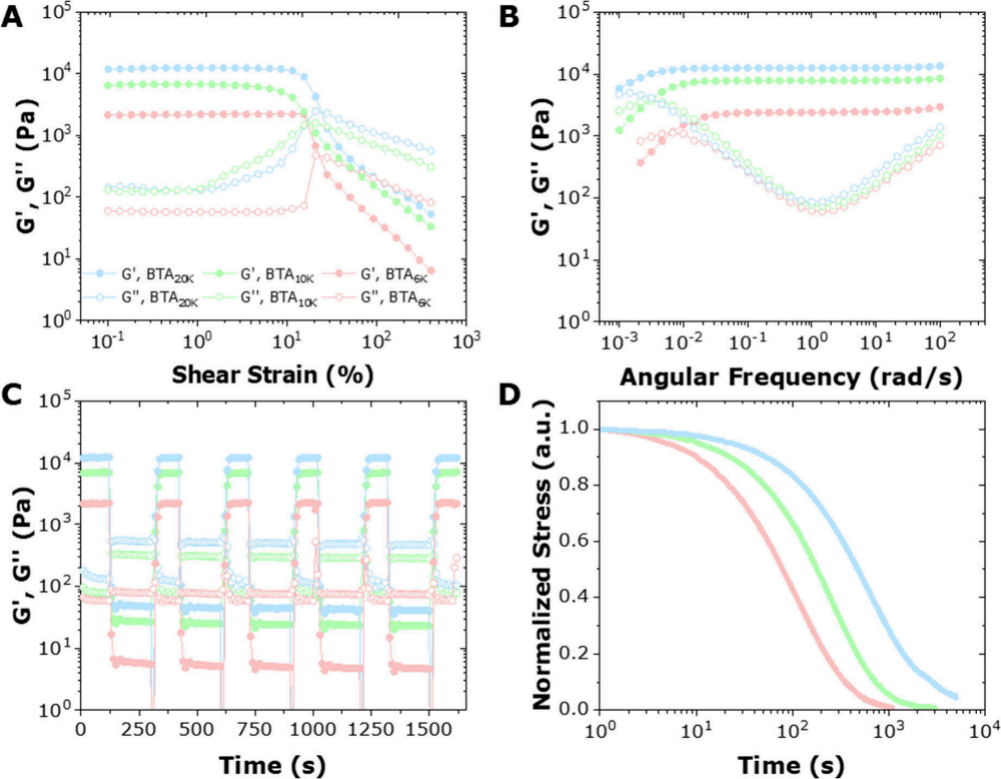
**Viscoelastic behavior of BTA hydrogels** A) In the amplitude sweep of BTA_6K_, BTA_10K_, and BTA_20K_, the higher molar mass BTA_20K_ has a significantly higher storage modulus (G′) with respect to the lower molar mass BTAs. B) The crossover-point found in the frequency sweep of all BTAs was shifted toward lower frequency regimes with increasing molar mass, suggesting that dynamics decrease with increasing PEG molar mass. C) Hydrogels are self-healing under shear rheology. Materials recover quickly (within seconds) to the initial G′ after rupture. D) Stress relaxation profile obtained by step-strain experiment at 1% strain of all three BTAs shows a higher *t*_1/2_ for higher molar mass BTAs. All hydrogels were measured at 10 wt %.

The differences in G′ across the series possibly suggest a dissimilarity in network cross-linking density. To exclude the effects of the 2.9-fold lower BTA concentration in the BTA_20K_ hydrogel than in the BTA_6K_ hydrogel at the same weight percent, BTA hydrogels with a constant BTA concentration (10 wt % BTA_20K_, 5 wt % BTA_10K_, and 3.3 wt % BTA_6K_) were created. Comparing their G′ would allow us to confidently isolate the impact of hydrophilic linker length on the viscoelastic behavior. Here, we found that G′ was significantly higher for BTA_20K_ (12.4 kPa) compared to BTA_10K_ (0.04 kPa) and BTA_6K_ (0.2 kPa) (Figure S23–24). These results further confirm our postulation that the PEG linker length is a key regulator of hydrogel stiffness in this supramolecular system. Comparing these data to PEG-only reference solutions would allow us to more directly separate polymer entanglement from BTA junction contributions and is considered an important follow-up for future dedicated studies.

As the materials were shown to be macroscopically self-healing (see above), all three formulations were subjected to a quantitative large-amplitude oscillatory shear experiment, ranging from low to high strain (1–400%), and the time for recovery was monitored (Figure 3C). All three hydrogels recovered their initial storage moduli almost instantaneously after structure breakdown, and this was observed for multiple cycles. Interestingly, these hydrogels maintained their viscoelastic behavior after storage at room temperature for over 28 days (Figure S25), indicating that these materials are stable and suitable for longer-term storage.

#### Internal Dynamics Dominated by PEG Linker Length

As observed from the frequency sweeps, the storage moduli of all three BTA hydrogels were largely frequency independent across almost five decades at 10 wt % (Figure 3B). The crossover between G′ and G′′ shifted to higher frequencies with decreasing PEG molar mass, reflecting faster internal dynamics. This trend aligns with Hafeez et al., who reported accelerated network dynamics and shorter relaxation times for systems with shorter outer hydrophobic spacers.^27^ Stress relaxation half-times, defined as the time needed for the sample to dissipate half of the initial stress, were used to benchmark dynamic behavior against literature and biologically relevant time scales.^13,14^ As shown in Figure 3D, *t*_1/2_ increased significantly with higher molar mass BTAs, from ≈80 s (BTA_6K_) to ≈180 s (BTA_10K_) and ≈480 s (BTA_20K_). Importantly, repeat measurements for 10 wt % formulations performed across independent batches and two rheometers showed that the trend was reproducible, with minor deviations in *t*_1/2_ (Figure S26A, B). Similar trends were observed upon increasing the total polymer content to 15 and 20 wt % (Table S3, Figure S27A, B). The correlation between increasing G′ and longer *t*_1/2_ may suggest that higher molar mass PEG linkers promote more interfiber bridging and entanglements, thereby restricting chain mobility and slowing down stress relaxation.^29,38–40^

#### Empirical Fitting Corroborates Experimental Impact of Linker Length on Stress Relaxation

The stress relaxation profiles of the BTA hydrogels followed a stretched exponential trend, suggesting multiple relaxation mechanisms could be at play (Figure 3D). To quantify this, the experimental data were fitted with a generalized two-element Maxwell model (Equation S1). This model accurately described the relaxation behavior across all formulations (Figure S28A–B; Table S4). The extracted parameters, namely amplitude (A), and characteristic short and long relaxation times (τ_1_ and τ_2_), respectively, revealed that increasing PEG molar mass and concentration led to overall longer relaxation times, particularly for τ_2_ (Table S5). Temperature-dependent stress relaxation experiments (20–50 °C) revealed a decrease in τ_1_ and τ_2_ with increasing temperature, consistent with thermally accelerated network dynamics (Figures S29–S30, Equation S2, Table S6). Notably, τ_1_ and τ_2_ exhibited parallel Arrhenius slopes for BTA_20K_, but diverging slopes for BTA_6K_, implying a different activation energy for the two relaxation modes in the shorter PEG system. This suggests a different interaction mechanism, possibly corresponding to the more enhanced bundling for this type, as seen in Figure 1. However, a direct correlation between each relaxation time and the corresponding microstructural processes is currently still elusive.

The hydrophilic linker length emerges as a key determinant of the hydrogels’ viscoelastic behavior by governing both intrinsic relaxation dynamics and thermal responsiveness. By adjusting PEG molar mass or concentration, stress relaxation time scales can be modulated by nearly an order of magnitude, balancing network elasticity and dynamics. A similar design principle is well established in ABA associative networks, ranging from classical triblock copolymers to telechelic supramolecular peptide–polymer conjugates. There, varying the midblock/PEG segment length modulates bridging probability and entanglement constraints, thereby shifting hydrogel stability/lifetime, the macroscopic plateau modulus, and relaxation spectrum.^41,42^ Notably, the BTA_6K_ 10 wt % exhibits *t*_1/2_ values within a biologically relevant range for mesenchymal stem cells (10–100 s), highlighting the system’s potential as tunable viscoelastic matrix for guiding cell mechanotransduction and fate.^4,6,13,43,44^ These observations mirror trends reported in other supramolecular systems. Nicolella et al. and Jangizehi et al. demonstrated that network heterogeneities, such as loops or junction clustering, can introduce multiple relaxation modes and, in some cases, accelerate stress dissipation by enabling internal stress redistribution.^45,46^ In contrast, Han et al. found that more uniform, bridge-rich networks exhibited slower and more constrained relaxation.^47^ These comparisons suggest that structural heterogeneity, when it promotes local mobility, can facilitate faster and multiscale stress relaxation.

### SAXS Analysis Reveals Differences in Network Architecture

Small-angle X-ray scattering (SAXS) analysis of these systems could further reveal the effect of PEG linker length on the hierarchical assembly. Here, we will focus on the impact of polymer chain length, concentration, and temperature. Real-space distances d correspond to observed scattering features and were calculated using Bragg’s law (Equation S3), enabling a more direct assignment of structural motifs.

SAXS spectra for all three BTA hydrogelators are given in Figures S31–33. Differences in concentration (10, 15, and 20 wt %) and temperature (20 or 37 °C) are highlighted in the spectra. The SAXS curves show four reproducible features that span the network hierarchy. Across BTA_6K_, BTA_10K_, and BTA_20K_ peak positions correlated with local BTA packing (q ≈ 1.1 and 0.6–0.7 nm^–1^) are nearly invariant, confirming hydrogen-bonded BTA stacks surrounded by the collapsed, hydrophobic C_12_ end chains are present in all cases.^48^ The diameter of these stacks is estimated to be around 10–12 nm, much larger than the estimate for single BTA molecules with C_12_C_12_ hydrophobic groups (d ≈ 5–6 nm), indicating multiple molecules per stacking layer. However, a systematic change in intensity and network length scale is observed. Here, (i) the relative intensity of BTA-related peaks decreases with increasing molar mass due to the decrease in BTA junctions per unit volume, (ii) the low-q mesh peak progressively moves to lower q (corresponding to real-space lengths of BTA_6K_ ≈ 18–23 nm, BTA_10K_ ≈ 19–25 nm, BTA_20K_ ≈ 22–25 nm). This is consistent with an increase in the characteristic mesh size, which we associate with a reduced cross-link density and longer PEG bridges. At the lowest q, BTA_6K_ further shows two (and weakly a third) broad correlation maxima with approximate peak ratios of q:√3 q:2q, suggestive of short-range hexagonal-like correlations. However, the peak breadth and low intensity prevent reliable indexing to a single hexagonal or cubic lattice. These low-q correlations weaken in BTA_10K_ and are further diminished in BTA_20K_, indicating that any mesoscopic ordering is strongest for the shortest PEG linker and decreases with increasing PEG length.

Overall, the topology seems to evolve from a BTA-bundle dominated, more dense and localized network (BTA_6K_), to a more porous, PEG–PEG entangled network (BTA_20K_), with BTA_10K_ lying in the middle ground. Similarly, an ABA triblock gel series shows that changing block lengths systematically shifts SAXS correlation features, reflecting altered mesoscale spacing and aggregate dimensions. This highlights that linker length can serve as a practical handle for tuning mesoscale organization in associative networks.^42^

Linking the rheology data to the SAXS data can help clarify why the BTA_6K_ (10 wt %, 20 °C) has a smaller network mesh size, yet a lower G′ and faster *t*_1/2_ compared to BTA_10K_ and BTA_20K_. In an ‘ideal’ transient network, the plateau moduli scale with the density of elastically active chains.^39^ However, since a significant fraction of chains can form loops instead of bridges, the network deviates from being ideal, lowering the number of stress-bearing chains.^46^ We believe that the smaller mesh size of BTA_6K_ observed in SAXS can be attributed to a more loop-enriched and short-bridged topology. In recent literature, similar results corroborate a consistent loop-induced decrease in G′, whereas bridge-enriched networks increase the hydrogels’ G′.^29,45,47,49^ As for the differences in *t*_1/2_, the increase observed in higher molar mass BTA hydrogels can be explained by the sticky-reptation behavior of longer PEG chains in the semidilute regime.^38,39^ There, the network is constrained so that the stress is dissipated only after 1) sticker exchange and 2) chain disengagement, leading to an increased *t*_1/2_ and raised G′, respectively. While quantitative fitting with sticky Rouse/reptation models could, in principle, yield junction lifetimes and estimates of the elastically active fraction, the absence of a clearly resolved terminal relaxation regime within our measured frequency window prevents robust, assumption-free fitting.

The combination of SAXS data and rheology possibly points toward a situation in which the BTA_6K_’s densely packed, yet loop-dominated topology relaxes more quickly. In contrast, the BTA_20K_ is organized in a more open, yet bridge- and entanglement-rich network leading to an increased G′ and *t*_1/2_. These findings align with prior analyses of associating polymer networks, which related micellar connectivity and bridging density to the resulting macroscopic viscoelasticity.^50^ However, more experiments would be needed to make definitive claims.

### Viscoelasticity and Stress Relaxation of BTA Hydrogel Blends

The fast relaxation time scale of the BTA_6K_ 10 wt % formulation is noteworthy, but its low stiffness makes it less suitable for use as a tissue engineering scaffold. More generally, viscoelastic hydrogel design often seeks to decouple mechanical integrity from relaxation dynamics, for example by incorporating network elements with distinct dynamic properties. For example, recent work by Xu et al. demonstrated that mixing supramolecular polymers with different kinetic lifetimes produces cooperative relaxation behavior.^51^ There, slower motifs effectively constrain the relaxation of faster motifs, broadening the stress relaxation spectrum while maintaining comparable plateau moduli. Building on this principle, we implemented a mixed hydrogelator strategy where the fast-relaxing, soft BTA_6K_ was combined with slower-relaxing, stiffer BTA_10K_ and BTA_20K_. Importantly, because these materials share the same BTA supramolecular motifs, this approach does not aim to create two independent, fully interpenetrating networks. Rather, it modulates the effective network dynamics through the composition of a single supramolecular network. By maintaining constant polymer content (10 wt %) and systematically varying the ratio of low- to high-molar mass BTAs, we aimed to investigate compositional effects on the hydrogels’ stiffness and internal dynamics.

#### BTA Blends Viscoelasticity

Frequency sweep plots for BTA_6:20K_ blends illustrate that the storage moduli fall fortuitously within similar regimes of their individual counterparts (Figure 4A, Table S7). BTA_6K,10K,20K_ hydrogel curves are replotted from Figure 3B and 3D to facilitate straightforward visual comparison. The frequency sweep data show that all blends act as viscoelastic solids in most of the probed frequency region, with a frequency-dependent behavior occurring below 0.1 rad/s. The 66:33 and 80:20 BTA_6:20K_ mixes have a stiffness (G′) lower than that of the pure 10 wt % BTA_20K_ hydrogel, but higher than the pure 10 wt % BTA_6K_. A similar relationship is observable for the G′-G′′ crossover point shift, leaning toward lower frequencies with increasing BTA_20K_ polymer contribution (66:33). Similar findings, i.e., an increase in G′ and extended G′–G′′ crossover point, were found for BTA_6:10K_ blends (Figure S34A). As anticipated for longer polymer chain lengths, the stiffness and G′–G′′ crossover point are higher for the BTA_6:20K_ blend than for the BTA_6:10K_ blend. However, the effect of increasing high *M*_w_ PEG (20 kg/mol) content from 80:20 to 66:33 on G′ appears less pronounced compared to the BTA_6:10K_ blend series. These observations reflect the composite impact of short and long chains on the material’s viscoelastic properties.

**Figure 4:**
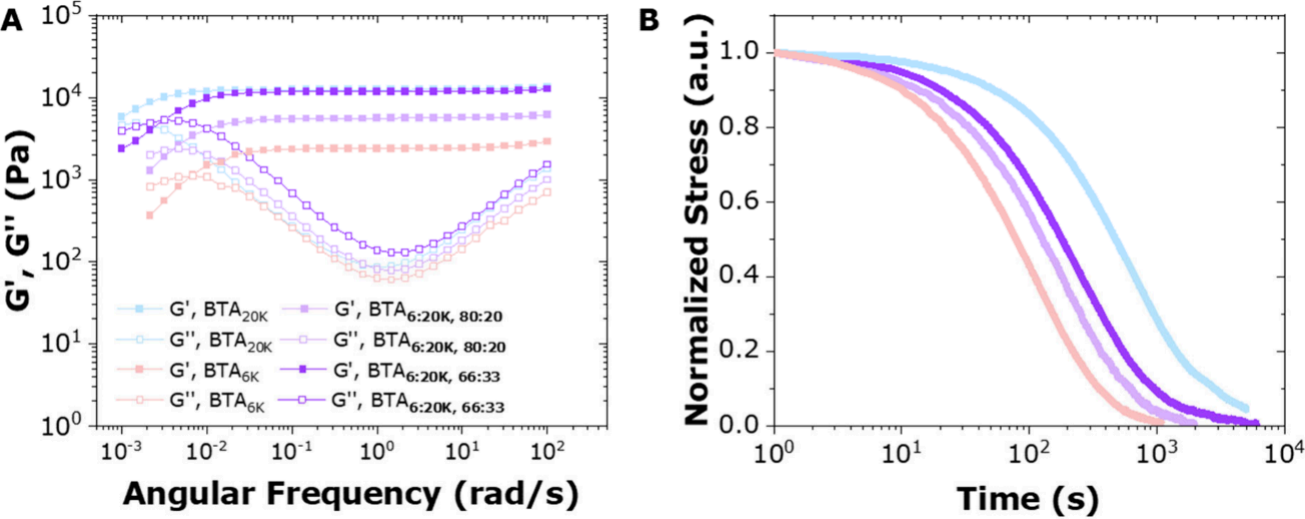
**Rheological characterization of blend-BTA supramolecular hydrogels**. A) frequency sweep of BTA_6K_ and BTA_20K_, alongside their respective 80:20 and 66:33 blends. G′ increases more strongly with higher BTA_20K_ content. B) Normalized stress relaxation profile for all four formulations at 1% strain. *t*_1/2_ shows a more modest increase with increasing BTA_20K_ content, less pronounced than the change in G′.

In parallel with the oscillatory shear rheology data, the stress relaxation curves depend on both (i) polymer length and (ii) blend ratio. The pure BTA_6K,10K,20K_ hydrogel curves from Figure 3B & 3D are replotted for easy visual comparison. Both tested blends (BTA_6:10K_ and BTA_6:20K_) produced intermediate relaxation kinetics (Figure 4B & S34B). Increasing the fraction of high *M*_w_ polymers from 20% to 33% generally leads to a linear increase in *t*_1/2_ (Table S8). This is supported by the G′-G′′ crossover-point shift to lower frequencies in all blend formulations, indicating slower internal dynamics. An increase in the number of longer polymer chains slows down relaxation (*t*_1/2_), whereas an increased fraction of shorter chains accelerates it. This is in line with the hypothesis that longer polymer segments hinder stress decay by constraining polymer mobility.

The tunability of mechanics and dynamics through mixing mirrors previous findings in supramolecular BTA systems, where combining monomeric and telechelic BTAs modulated the network’s behavior.^29^ However, for the specific compositions investigated, blending produced a largely monotonic and approximately proportional dependence of G′ on composition without synergistic enhancement or nonmonotonic response. This more additive behavior contrasts with that of Vlassopoulos et al., in which mixing different supramolecular species led to nonmonotonic mechanical trends similar to that of wormlike micellar mixtures.^29^ In this context, our blend results are intended as an exploratory perspective, demonstrating a simple route to tune viscoelasticity (G′ and *t*_1/2_) by adjusting composition, rather than establishing general mixing rules for supramolecular networks. A systematic study varying blend ratio, interaction strength, and molecular architecture will be needed to delineate when nonmonotonic responses emerge, an important direction for future work.

### Extrusion-Based Printing of BTA Hydrogels

#### Shear Thinning, 3D Printing, and Zonal Scaffolds

Through steady shear rheology, the effect of linker length molar mass on viscosity was examined in 10 wt % hydrogels (Figure 5A). An increase in shear rate was observed to decrease the viscosity for all three BTA hydrogelators, indicating shear thinning behavior. This can be rationalized by the rapid exchange of BTA molecules between fibers. Increasing the molar mass of the linker segments was observed to increase the consistency index (K) and modulate flow index (n) (Table S9). Simultaneously, steady-shear flow curves at 10 wt % show that shear stress at a given shear rate increases systematically with PEG linker length (BTA_6K_ < BTA_10K_ < BTA_20K_), indicating that BTA hydrogelators with longer linker lengths form more robust, flow-responsive networks (Figure S35).

**Figure 5:**
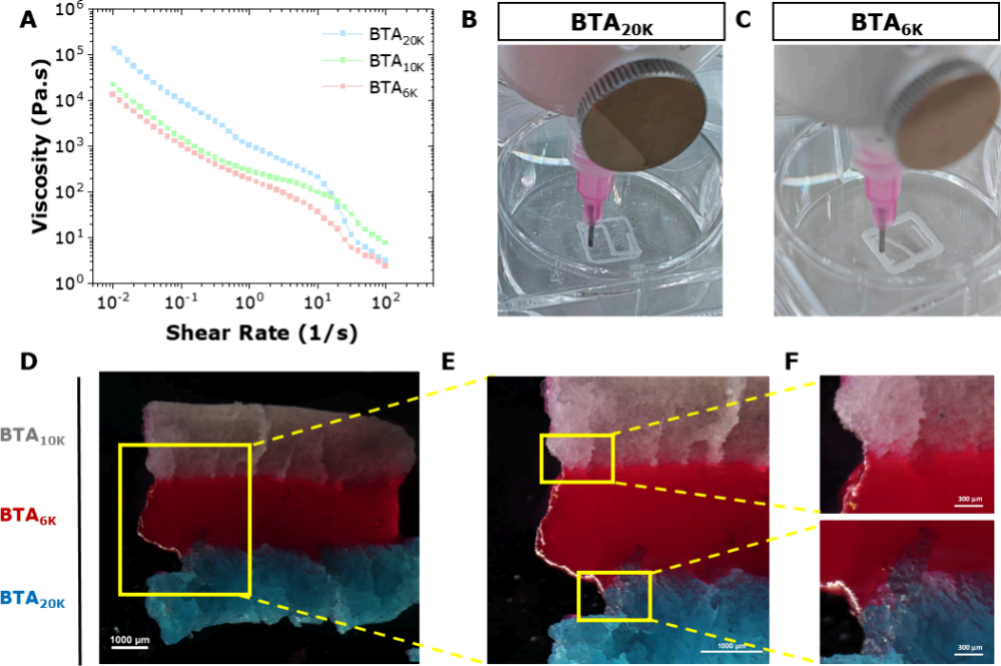
**Shear thinning and extrusion 3D printing of BTA hydrogels**. A) Decrease in viscosity with increasing shear rate showed shear-thinning behavior of all BTA hydrogels (10 wt %). B) Extrusion-based printing of BTA_20K_ monolayers and C) BTA_6K_ *z*-axis stacked fibers. D) Extruded filaments of BTA_10K_ (gray), BTA_6K_ (red), and BTA_20K_ (blue) hydrogels in parallel. E, F) Increased magnification shows hydrogels are visually inseparable and have self-healed at their interfaces.

Given their self-healing and shear-thinning behavior, BTA_20K_ and BTA_6K_ hydrogels were printed at 10 wt % using an extrusion-based BioX 3D printer on a 48-well plate (Figure 5B, C). All hydrogels were extruded as continuous and uniform filaments, allowing for stacking, which provided a baseline for printing scaffolds with complex architectures. All structures retained their shape after printing, even when the well plate was inverted. Notably, the BTA_20K_ required the highest pressure for extrusion (200 kPa) into filaments, compared to 70 kPa required for BTA_6K_. This increase in extrusion pressure can be rationalized by the apparent increase in viscosity observed with longer linker lengths at the relevant shear rates (1–10 1/s). These findings align with the molar-mass-driven variation of consistency index (K) and the flow indices (n) described above. Additionally, physical manipulation of the materials showed increased fiber fragility with increasing molar mass. Although extrusion of 10 wt % BTA hydrogels requires relatively high pressures that can be suboptimal for cell-laden bioprinting, this limitation can be mitigated by lowering total polymer content, thereby reducing bioink viscosity and required extrusion pressure into a more cell-friendly regime.^52^

Biological tissues rarely display uniform properties, particularly at their interface. Instead of sharp boundaries, smooth gradients support mechanical and biological functions. Cartilage is an example of a material with zonal stiffness increasing progressively from the soft, superficial zone to the calcified deep zone. Inspired by this natural, hierarchical complexity, we aimed to develop a construct containing zonal gradients in stiffness and stress relaxation. To achieve this, BTA hydrogels (10 wt %) were extruded manually through a syringe, allowing parallel deposition of filaments. Hydrogels were colored using food-grade dyes, which were not found to noticeably alter G′ or the CGC for any BTA hydrogelator. Figure 5D shows the individual BTA filaments combined into a large scaffold. The construct was found to be self-supporting and maintained cohesion while being manipulated with tweezers (Figure S36). Focusing on the hydrogel interfaces (Figure 5E), it becomes evident that all BTAs self-heal between layers and adhere smoothly to each other, with no visible separation. This way, we demonstrate that the hydrogels are visually indistinguishable at their interface and yield a construct with spatially determined mechanical properties due to the ability of the BTA gels to self-heal with one another.

Previous efforts to establish zonal gradients with BTA hydrogels have relied on covalent cross-linking strategies.^53^ Here, norbornene functionalities were introduced, enabling thiol–ene cross-linking. This allows modulation of the hydrogel’s stiffness by adjusting the thiol:norbornene ratio, leading to the fabrication of complex scaffolds with spatially defined viscoelasticity. While this method can produce hydrogels with gradient mechanics, it permanently immobilizes the polymers, restricting network dynamics. To our knowledge, this approach resulted in the first supramolecular hydrogel with gradient viscoelasticity generated exclusively through supramolecular interactions. By removing the requirement of covalent cross-linking, this platform maintains the dynamic, reversible nature of supramolecular scaffolds and offers a biomimetic route for developing tunable zonal architectures.

## Conclusion

In this work, we synthesized a library of BTA hydrogelators with varying hydrophilic PEG linker length. Through CryoTEM and self-assembly studies in dilute solution, we found that morphologies are largely independent of PEG molar mass. All BTA hydrogelators formed stable hydrogels at 10 wt %, with the CGC being inversely related to PEG molar mass. Through rheological measurements we identified that G′ and *t*_1/2_ depend on PEG linker length, where higher molar mass leads to stiffer, and slower relaxing networks. This trend in G′ directly opposes the findings for covalent systems, challenging the original hypothesis. These findings were corroborated by X-ray scattering data, which indicated a smaller mesh size and suggested increased looping of BTA_6K_ compared to BTA_20K_. In contrast to our expectations, blending high- and low-molar-mass BTA hydrogelators did not synergistically enhance the stiffness. All hydrogels were injectable and showed good shape fidelity after extrusion 3D printing, where a decrease in PEG linker length facilitated easier extrusion. Constructing a zonal scaffold using exclusively supramolecular interactions showed adhesion and fusion of the various BTA hydrogels at the interface. We demonstrate the importance of developing a BTA hydrogelator library and its perks for creating scaffolds with tunable viscoelasticity. In the future, this library can be used to elaborate on the impact of supramolecular scaffold viscoelasticity on relevant cell lines, extending their application potential even further.

## Supplementary Material



## Notes

Additional ^1^H- and ^13^C NMR spectra; additional GPC experiments; additional Nile Red experiments; additional DLS experiments; additional CryoTEM images; additional rheology experiments; additional SAXS measurements; additional photographs of hydrogels (PDF)
