# Supplementary material for: Linker Molar Mass-Driven Control over Supramolecular Network Relaxation and Architecture in BTA Hydrogels
Source: Macromolecules. 2026 Jul 10;59(14):8375–87. doi: 10.1021/acs.macromol.5c03492 (PMC13421974; doi:10.1021/acs.macromol.5c03492)
Supplement: Supplementary file 1 [file ma5c03492_si_001.pdf]

Supporting Information for

Linker molar mass-driven control over supramolecular network relaxation and architecture in BTA hydrogels

Arthur Helsen[1, 2, 3], João S. Ribeiro[3], Ivo A. Beeren[3], Hans Duimel[5], Ruth Cardinaels[4], Lorenzo Moroni[3], Louis M. Pitet*[2], Matthew B. Baker*[1,3]

[1] Department of Instructive Biomaterials Engineering, MERLN Institute for Technology Inspired Regenerative Medicine, Maastricht University, P.O. Box 616, 6200 MD Maastricht, the Netherlands

[2] Advanced Functional Polymers Group, Department of Chemistry, Institute for Materials Research (IMO), Hasselt University, Martelarenlaan 42, 3500 Hasselt, Belgium

[3] Department of Complex Tissue Regeneration, MERLN Institute for Technology Inspired Regenerative Medicine, Maastricht University, P.O. Box 616, 6200 MD Maastricht, the Netherlands

[4] Soft Matter, Rheology and Technology, Department of Chemical Engineering, KU Leuven, Celestijnenlaan 200J, 3001 Leuven, Belgium

[5] Maastricht MultiModal Molecular Imaging Institute, Maastricht University, P.O. Box 616, 6200 MD Maastricht, the Netherlands

*Email corresponding authors: m.baker@maastrichtuniversity.nl; louis.pitet@uhasselt.be

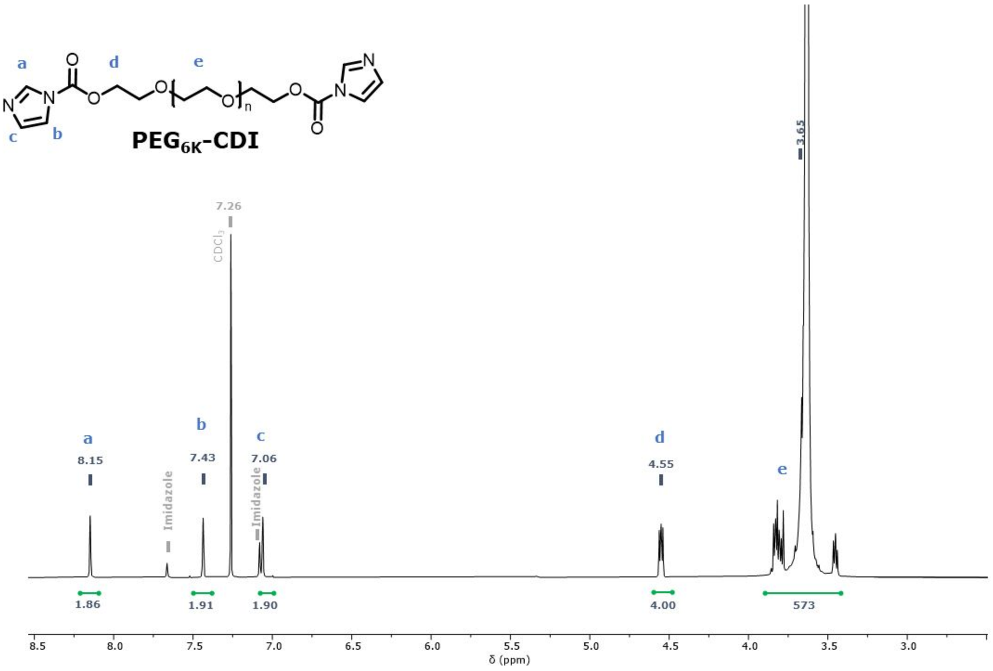

Figure S1: ^{1}H-NMR PEG$_{6K}$-CDI in $CDCl_3$

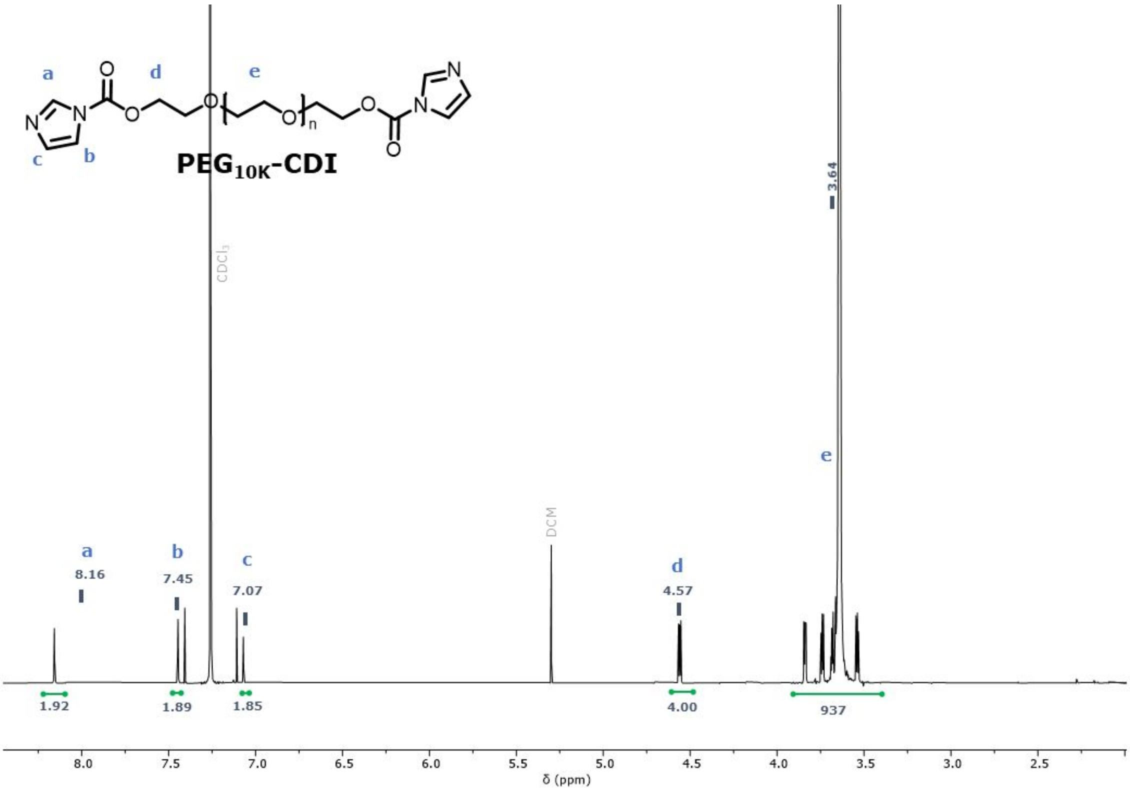

Figure S2: ^{1}H-NMR PEG_{10K}-CDI in $CDCl_3$

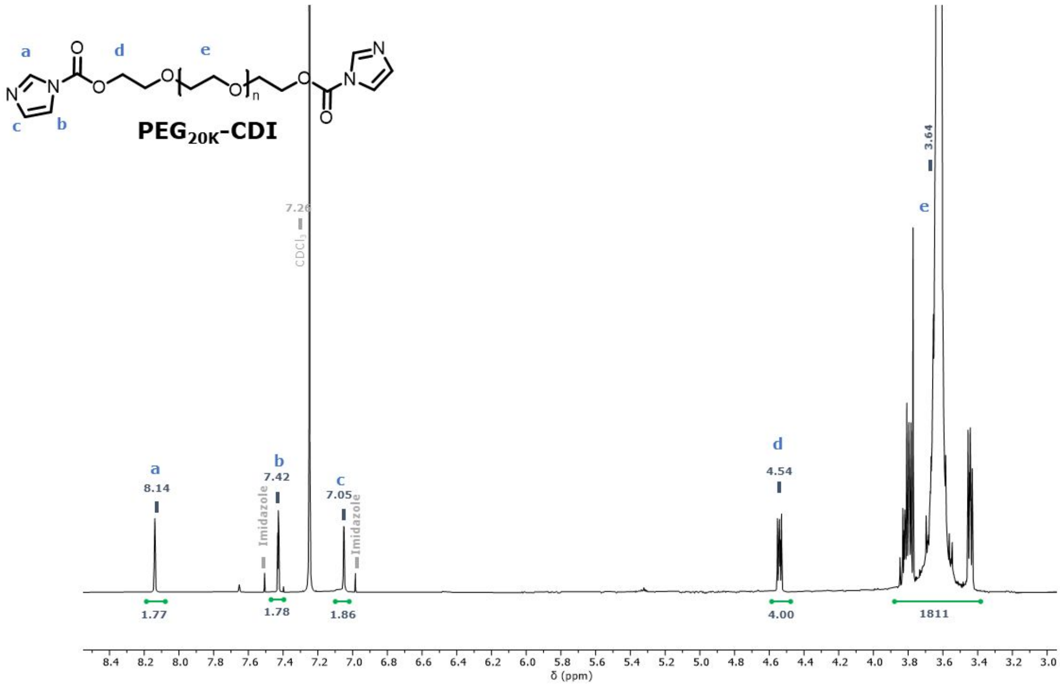

Figure S3: ^{1}H-NMR PEG_{20K}-CDI in $CDCl_3$

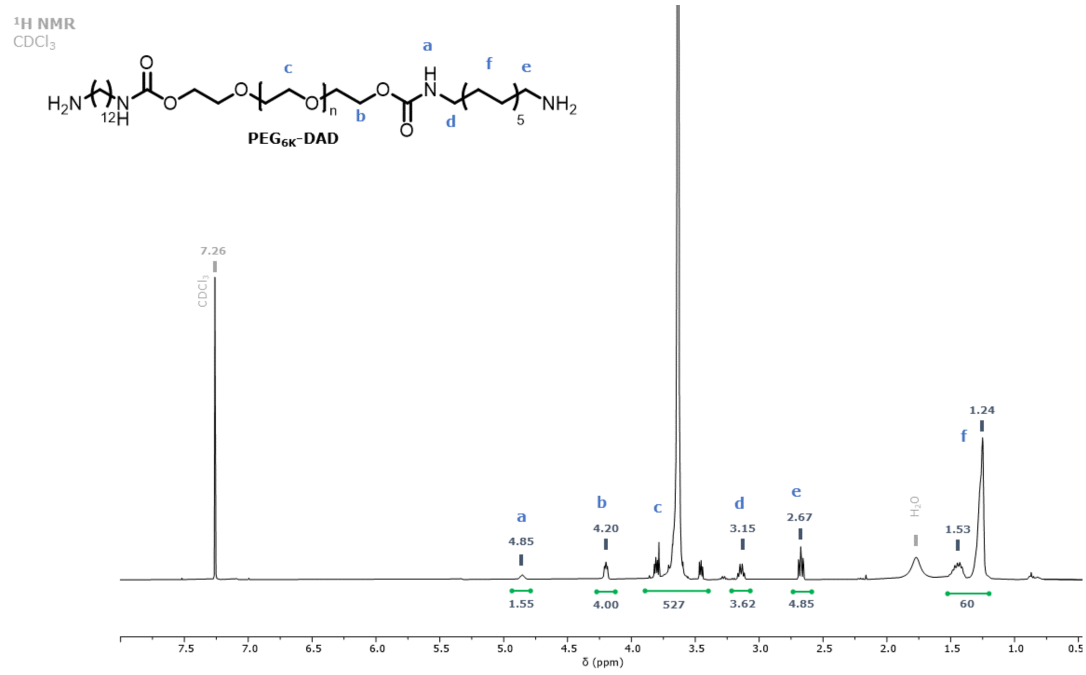

Figure S4: ^{1}H-NMR PEG$_{6K}$-DAD in $CDCl_3$

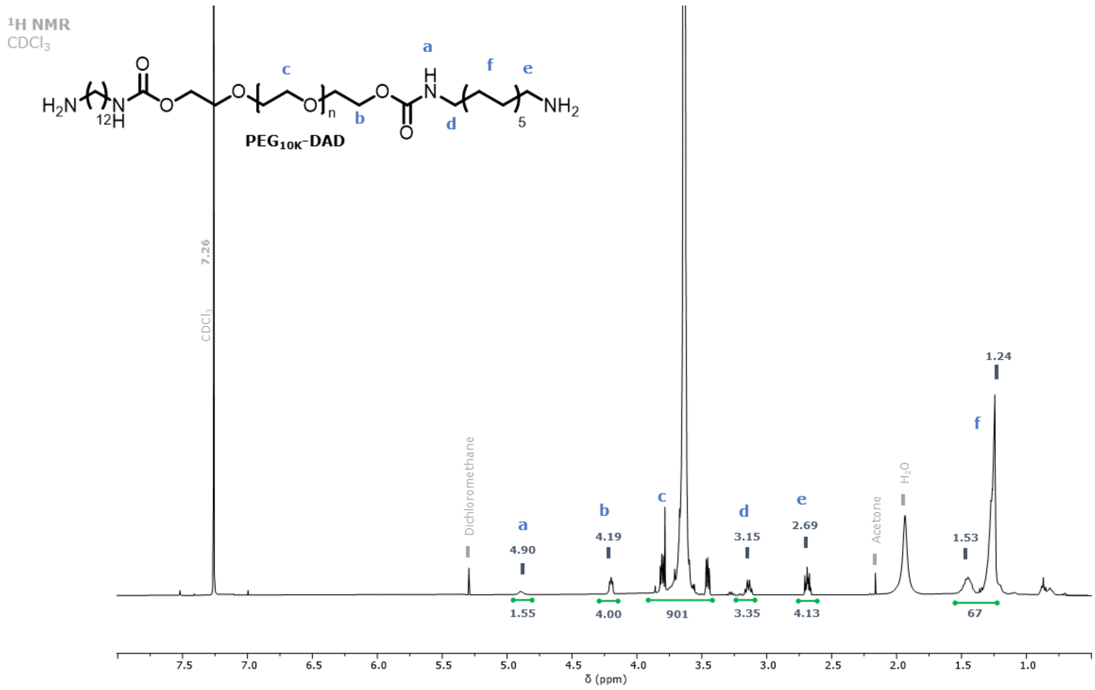

Figure S5: ^{1}H-NMR PEG$_{10K}$-DAD

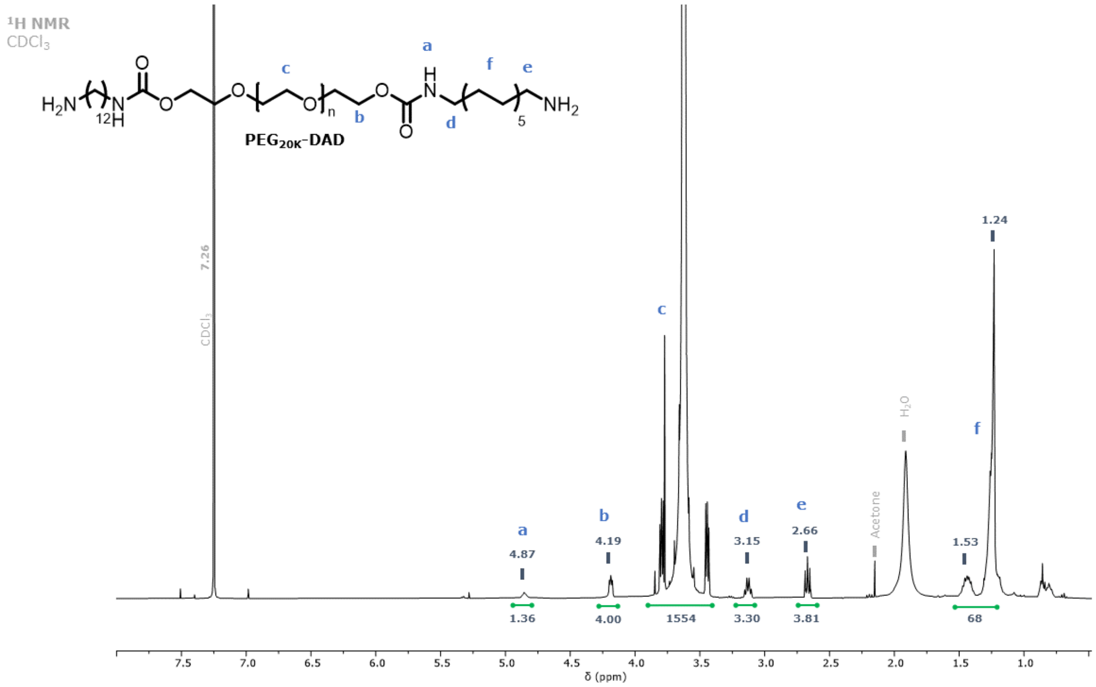

Figure S6: ^{1}H-NMR PEG$_{20K}$-DAD in CDCl$_3$

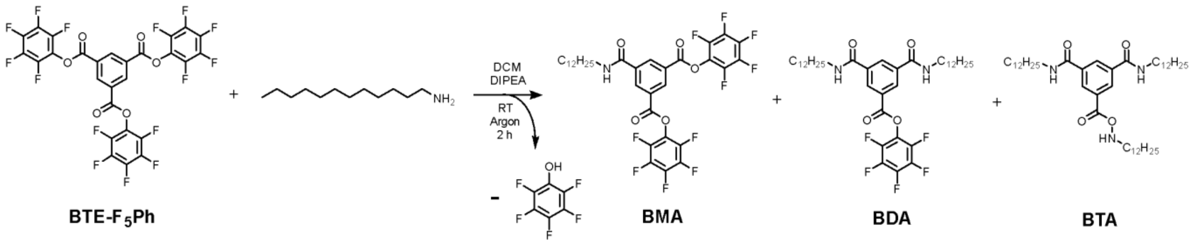

Scheme S1: Desymmetrization reaction from symmetrical BTE-F$_5$Ph synthon. BMA = Benzene-1-monocarboxylicacid; BDA = benzene-1,3-dicarboxylicacid; BTA = benzene-1,3,5-tricarboxylicacid

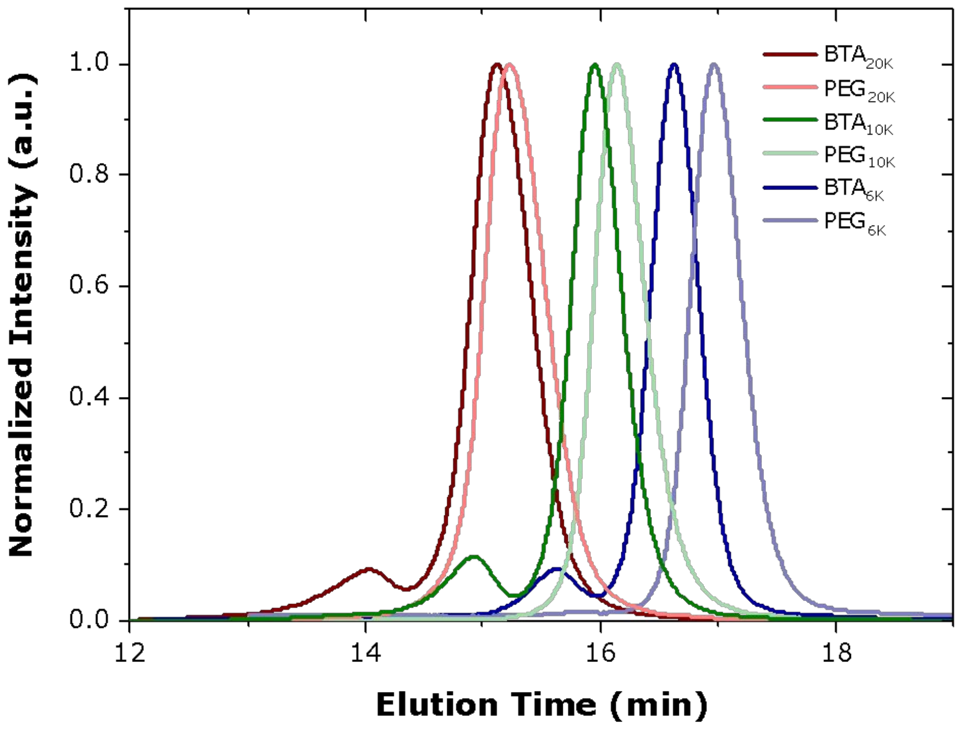

Figure S7: SEC eulogram of synthesized polymers and their PEG precursors. Peaks at ± 15% intensity correspond to chain-extended products.

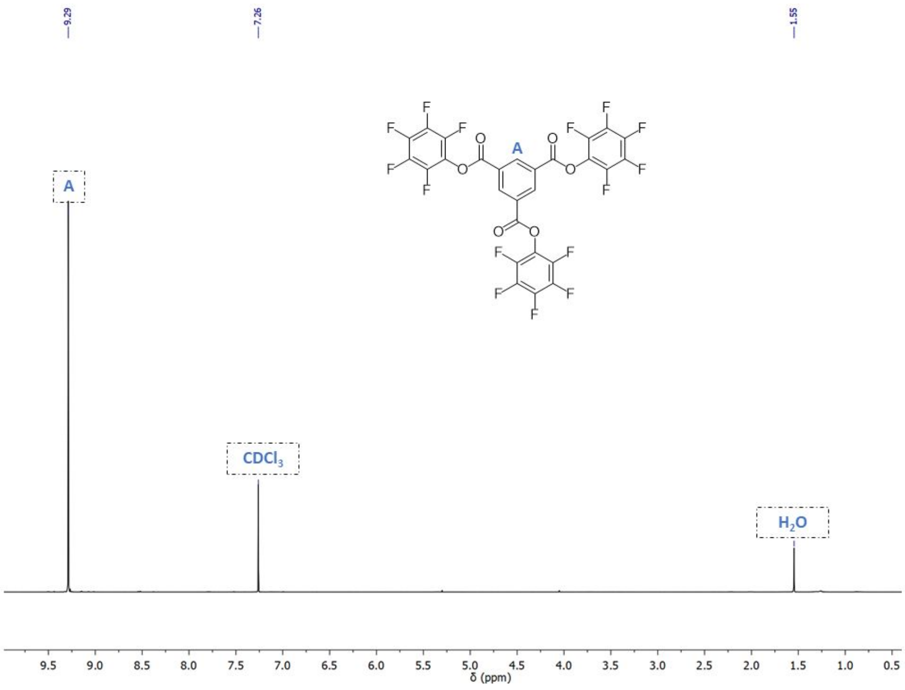

Figure S8: ^1H-NMR of BTE-F_5Ph in $CDCl_3$

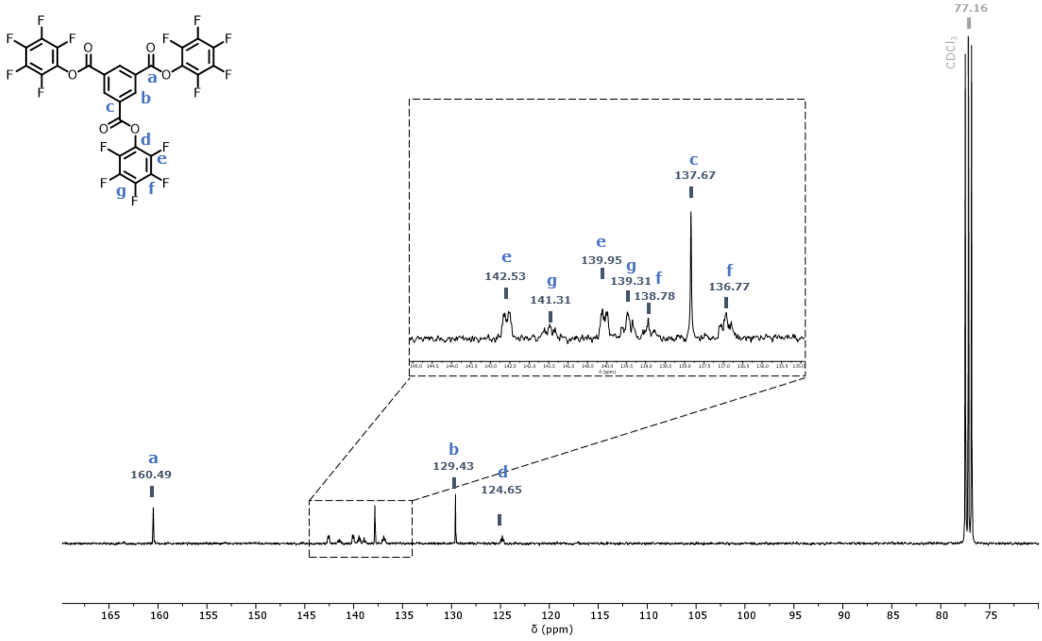

Figure S9: ^{13}C-NMR of BTE-F_5Ph in $CDCl_3$

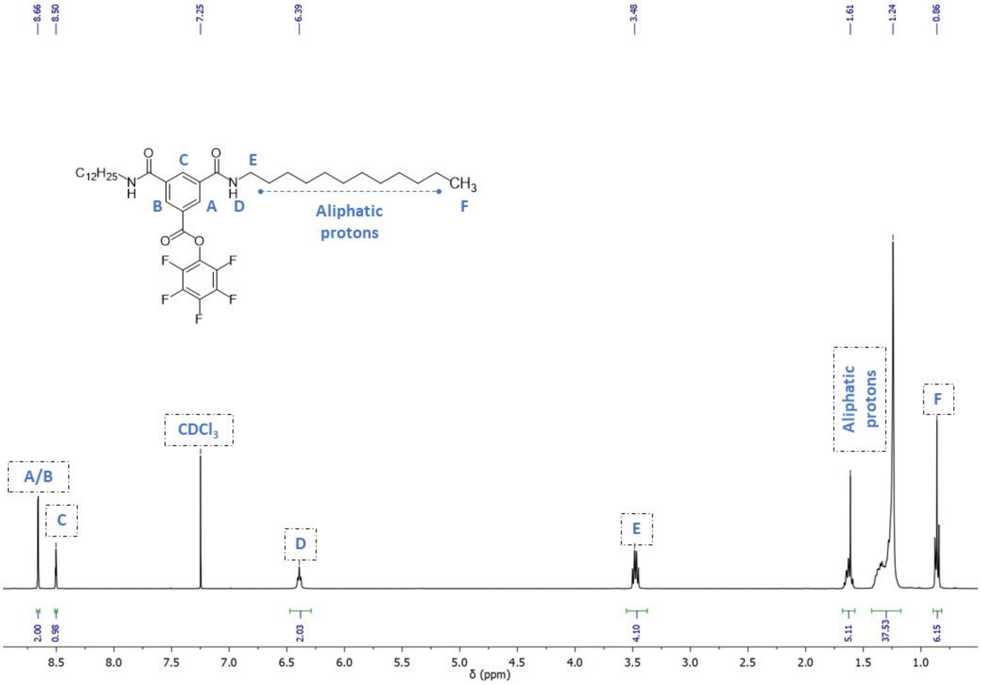

Figure S10: ^{1}H-NMR of BDA_{C12} in $CDCl_3$

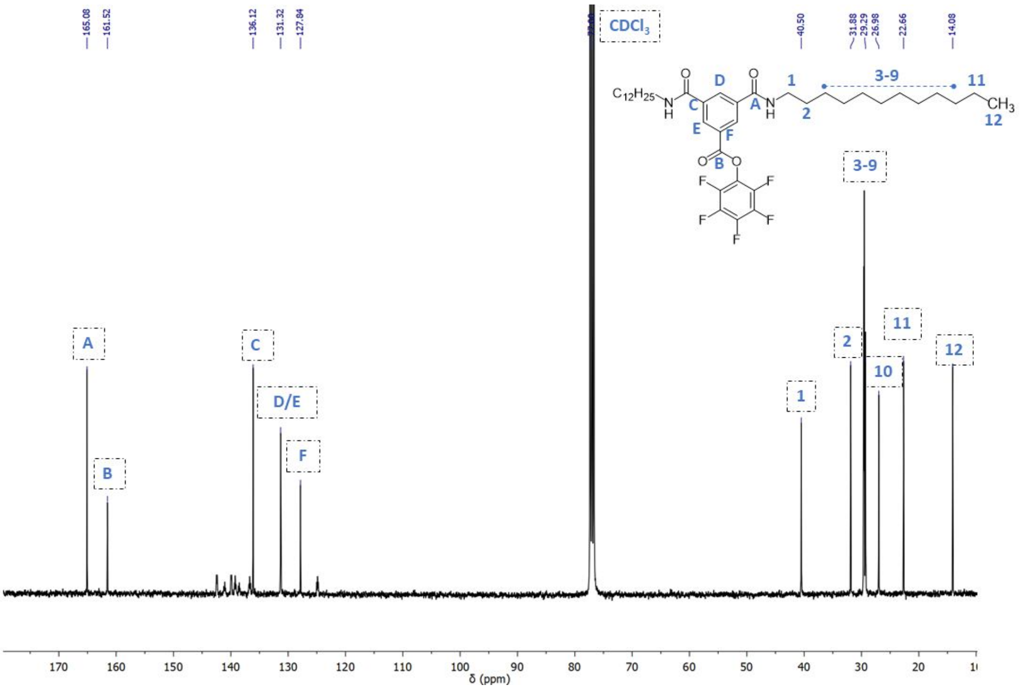

Figure S11: ^{13}C-NMR of BDA_{C12} in $CDCl_3$

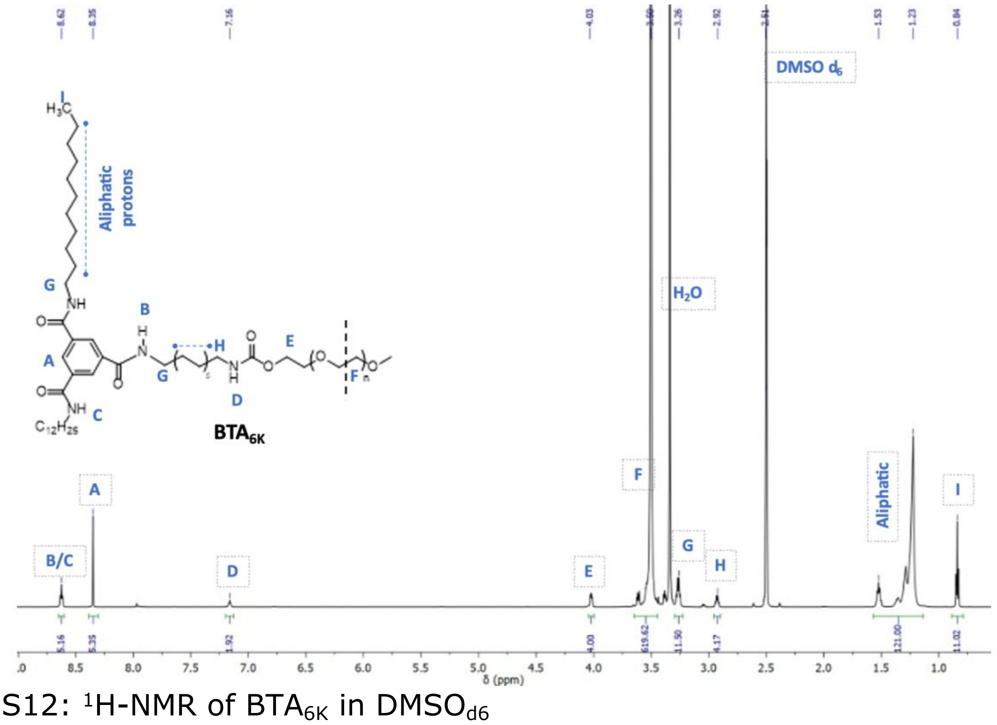

Figure S12: ^{1}H-NMR of BTA_{6K} in $DMSO_{d6}$

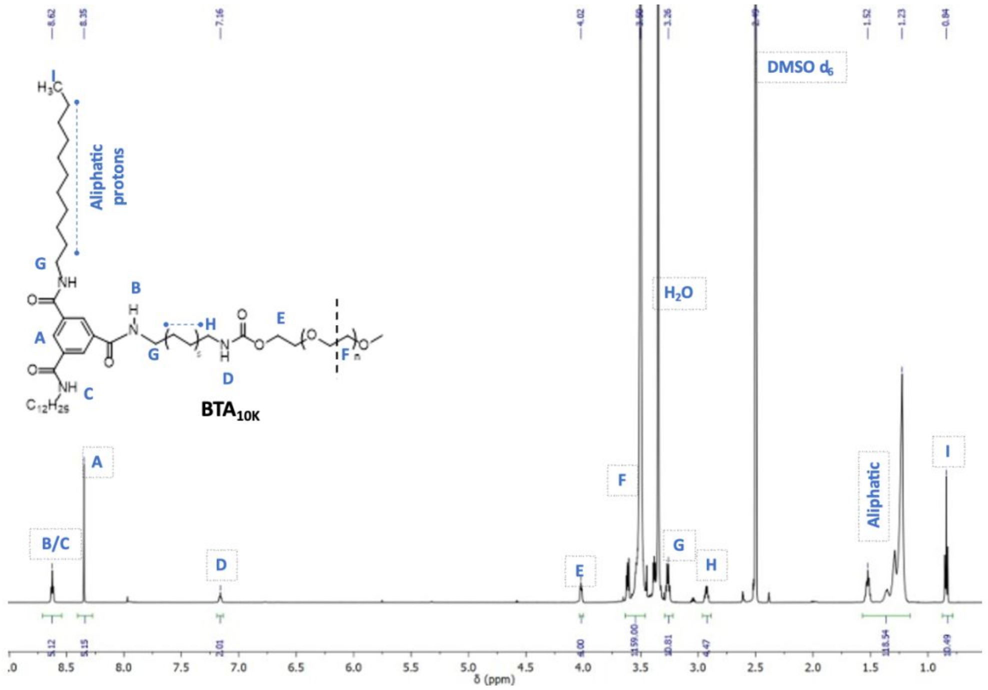

Figure S13: ^{1}H-NMR of BTA_{10K} in $DMSO_{d6}$

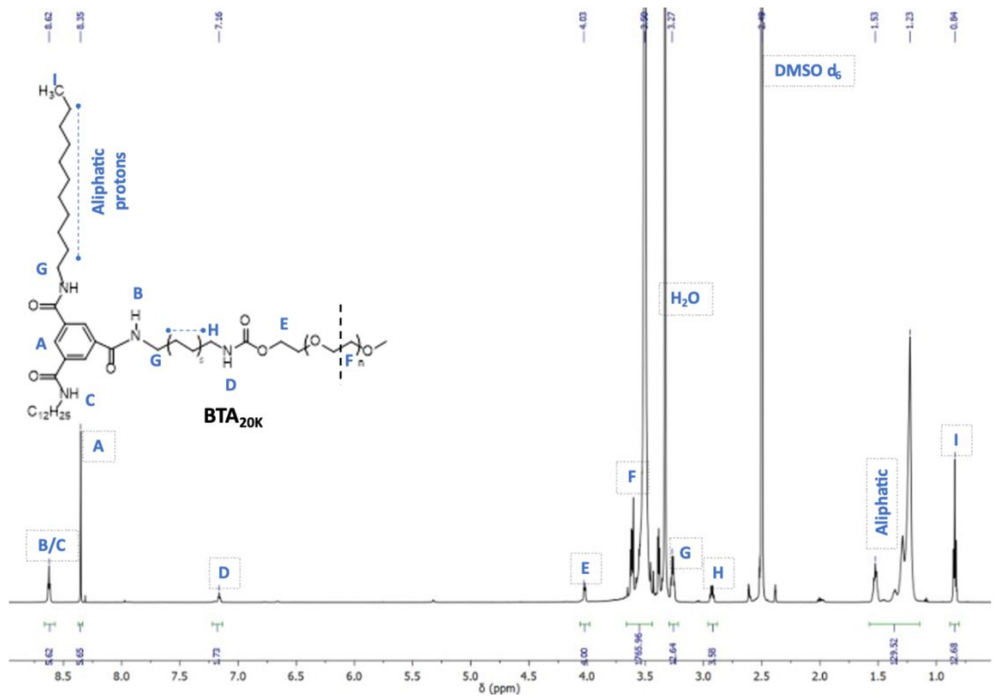

Figure S14: ^{1}H-NMR of BTA_{20K} in $DMSO_{d6}$

Table S1: GPC data for BTA macromolecules (in $CHCl_3$)			
	M_w (kg/mol)	M_n (kg/mol)	$Đ$ (M_w/M_n)
BTA_{6K}	17.7	15.8	1.12
BTA_{10K}	30.1	26.5	1.13
BTA_{20K}	57.7	49.1	1.18

Table S2: MALDI-TOF m/z data for BTA hydrogelators			
	BTA_{6K}	BTA_{10K}	BTA_{20K}
**m/z**	7941.793	12053.136	23157.007

Nile Red

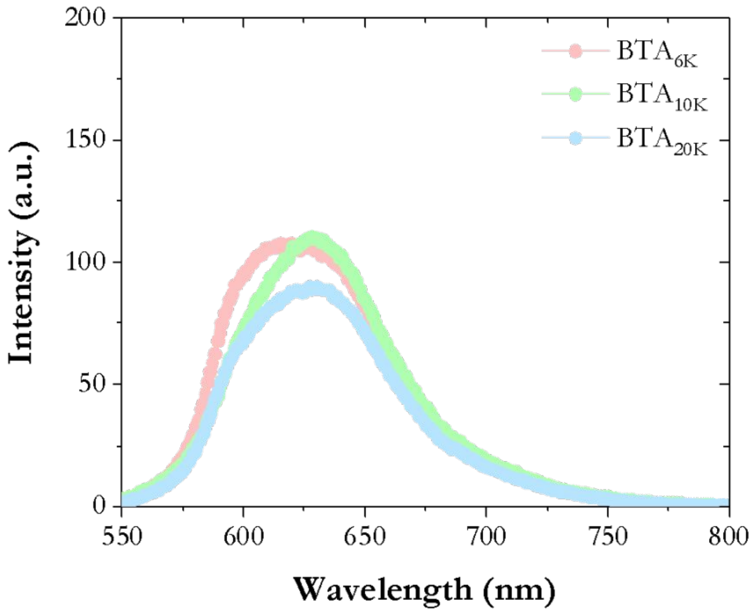

Figure S15: Emission spectrum (ex = 540 nm) of Nile Red in all three dilute BTA macromolecule solutions at 50 μM [BTA].

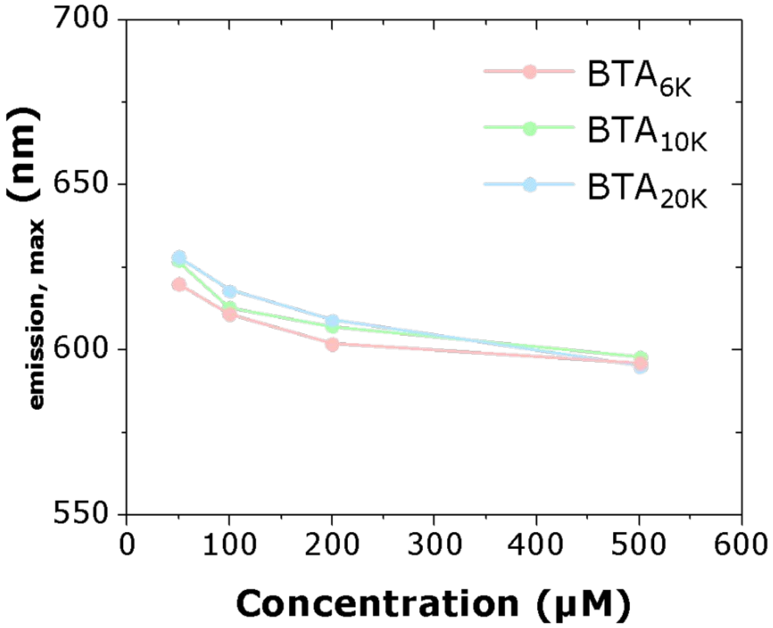

Figure S16: Maximum emission wavelength for all BTAs at various concentrations shows blueshift independent on PEG molar mass.

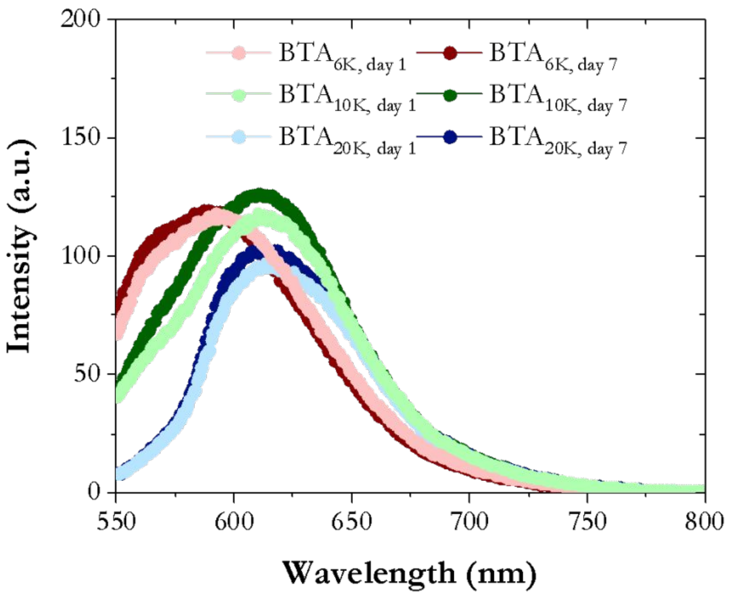

Figure S17: The overlap of Nile Red fluorescence emission spectra after 1 day (light colors) and 7 days (dark colors) indicates that supramolecular assemblies remain constant over time. Conc. = 5 mg/mL

CryoTEM

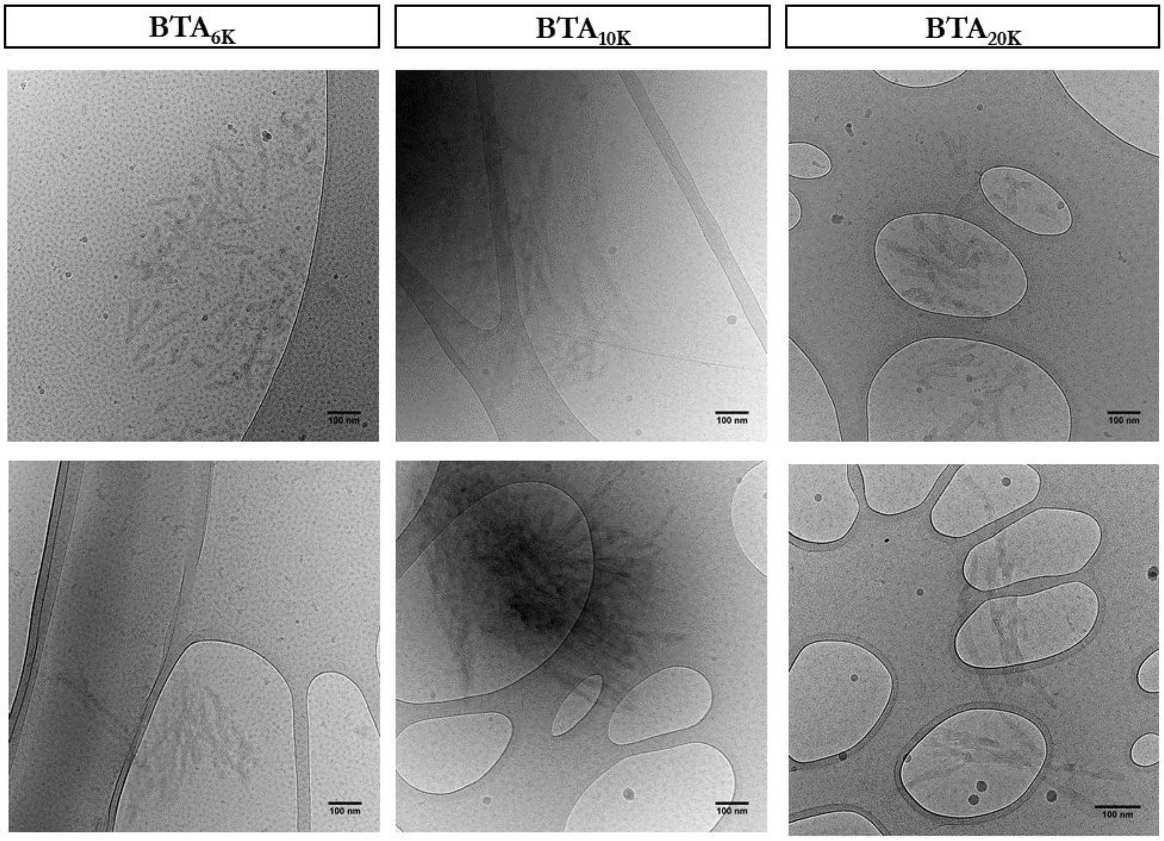

Figure S18: Self-assembly studies using CryoTEM. $BTA_{6,\ 10,\ 20K}$ diluted solutions in water:methanol (95:5). All BTAs show fibrous aggregates. Conc. $BTA_{6K/10K}$ = 10 mg/mL; Conc BTA_{20K} = 5 mg/mL

Dynamic Light Scattering

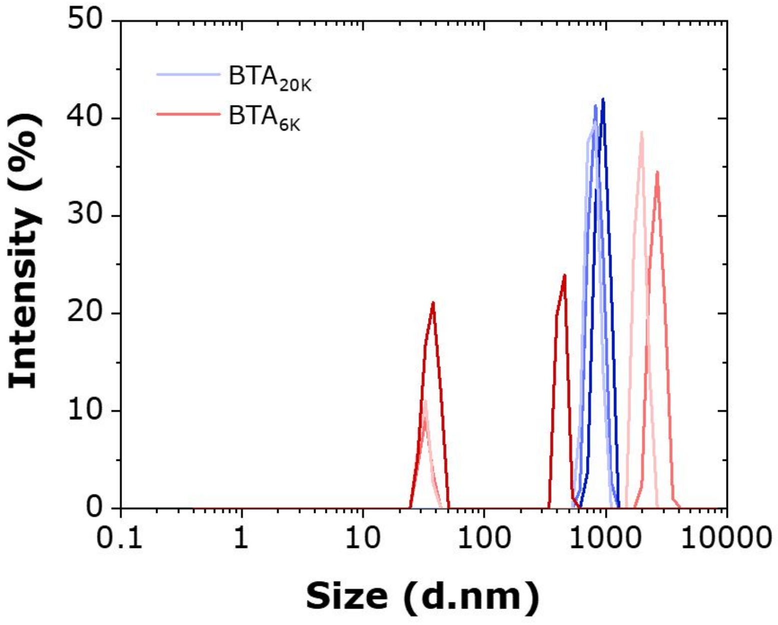

Figure S19: Dynamic Light Scattering (DLS) intensity-weighted size distribution for BTA_{20K} (blue shades) and BTA_{6K} (red shades) at 10 mg/mL. For each sample, three consecutive measurements were performed, represented by a change in color shade.

Rheology

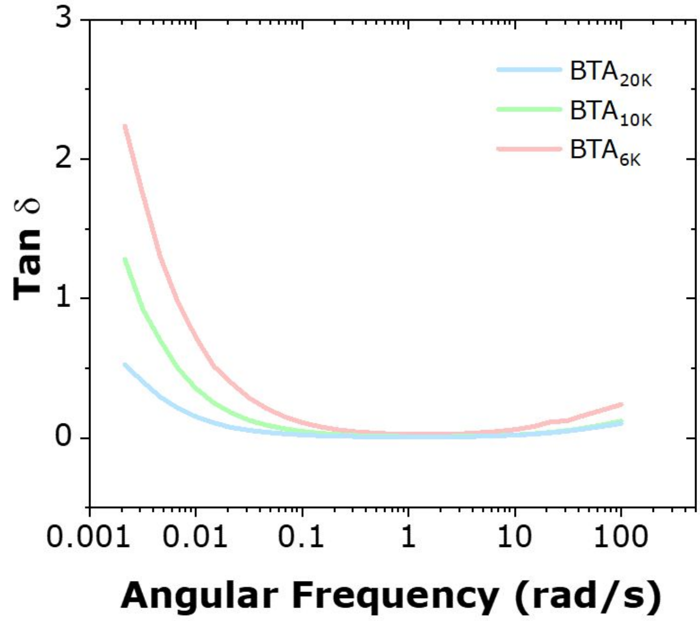

Figure S20: Frequency dependence of tan δ for 10 wt % BTA hydrogelators measured by oscillatory shear rheology. For all formulations, tan δ remains < 1 (G′ > G′′) over the relevant frequency range, confirming predominantly elastic, gel-like behavior at 10 wt %.

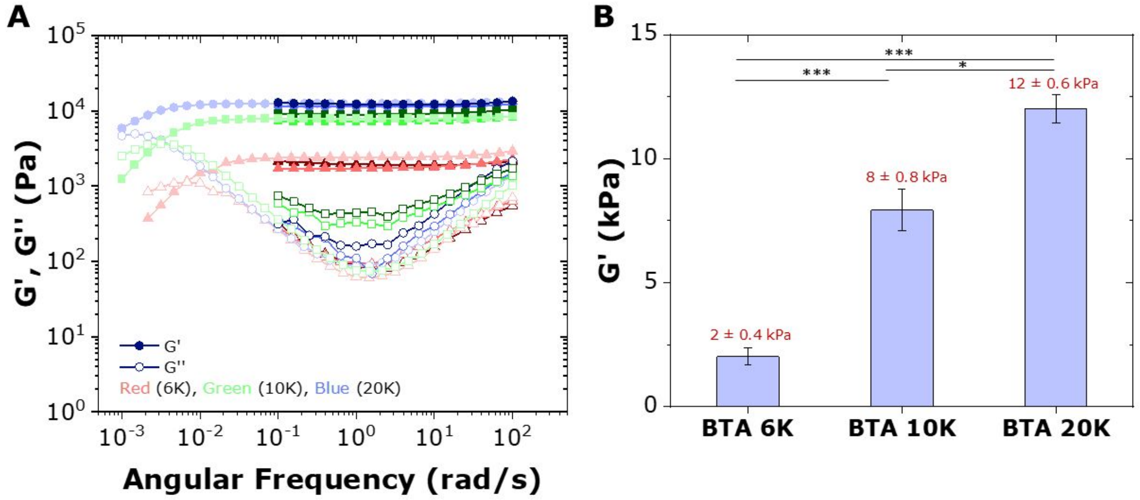

Figure S21: Repeat frequency sweep measurements. A) Measurements performed on HR20 (TA Instruments) for BTA_{6K}, BTA_{10K}, and BTA_{20K} (dark shades) overlaid with the corresponding data (light shade) from Figure 3B in the main text (Anton Paar MCR702). B) G′ at 1 rad/s shown as mean ± SD for the repeated measurements (n = 3). The similar values obtained across independent measurements indicate good reproducibility across batches, instruments, and measurement locations. Differences in G′ based on linker length were significant (*p < 0.05, ***p < 0.001).

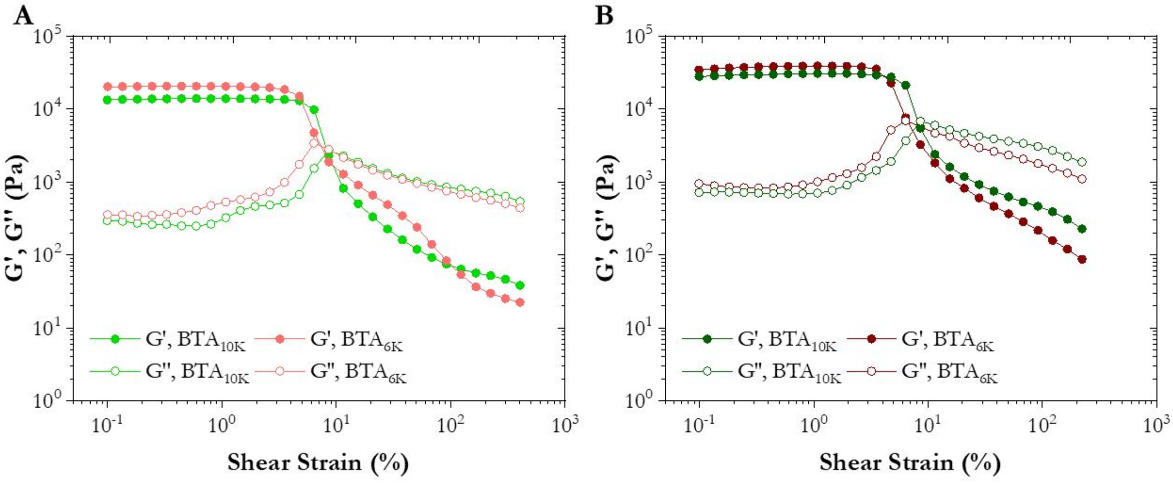

Figure S22: Amplitude sweeps of BTA_{6K} (red) and BTA_{10K} (green) hydrogels at A) 15 wt % and B) 20 wt %.

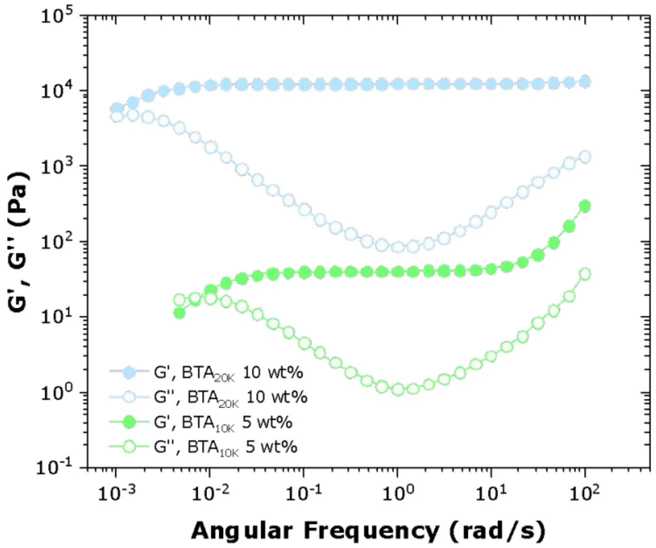

Figure S23: Frequency sweeps from 10 wt % BTA_{20K} and 5 wt % BTA_{10K} hydrogels

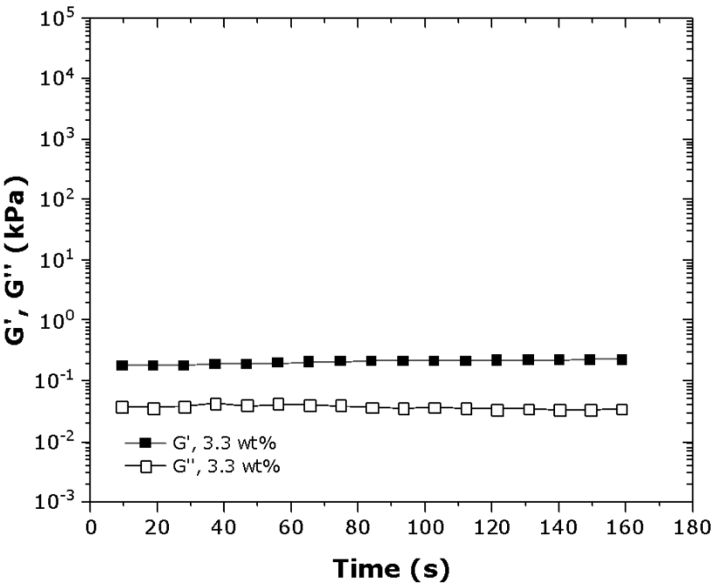

Figure S24: Time sweep of BTA_{6K} 3.3 wt % at 1% strain and 1 rad/s.

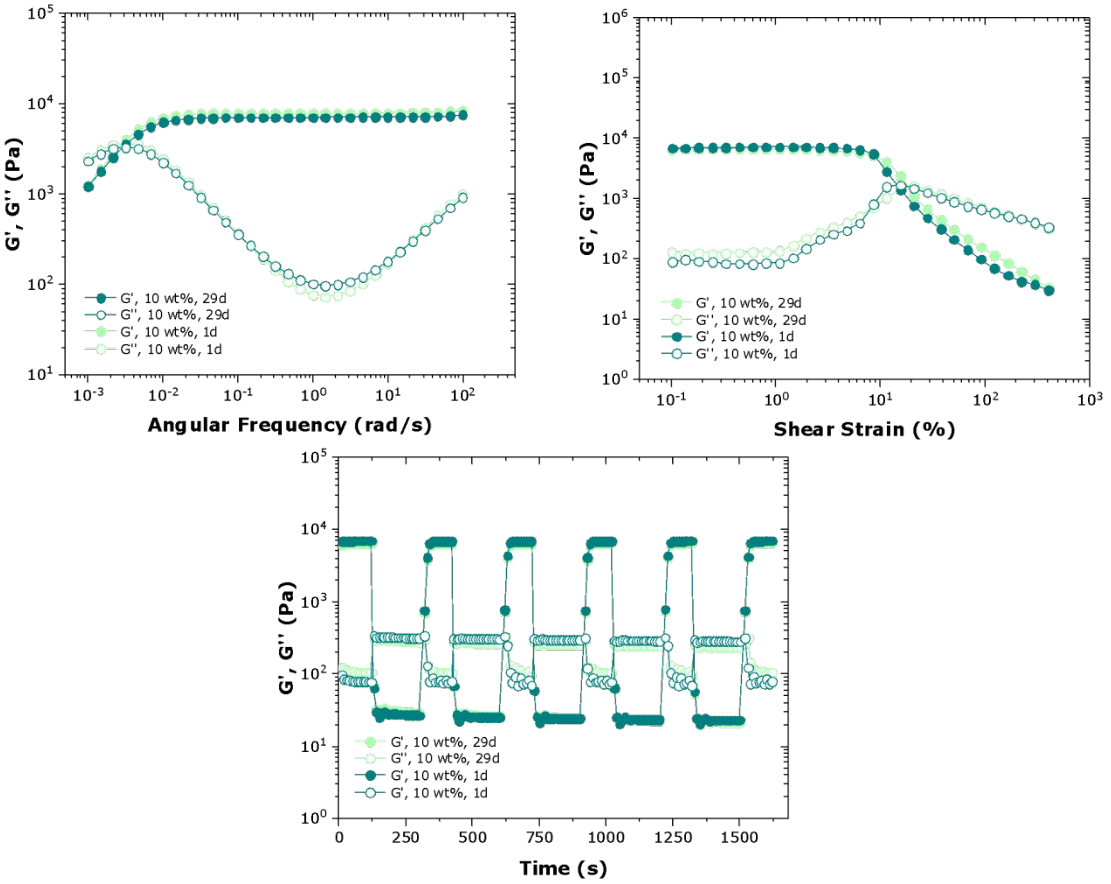

Figure S25: Viscoelastic characterization of 10 wt % BTA_{10K} hydrogels at day 1 (light green) and day 29 (dark green). A 500 µL hydrogel was prepared and split in half, where each half was measured at the mentioned timepoints. The material was stored at room temperature.

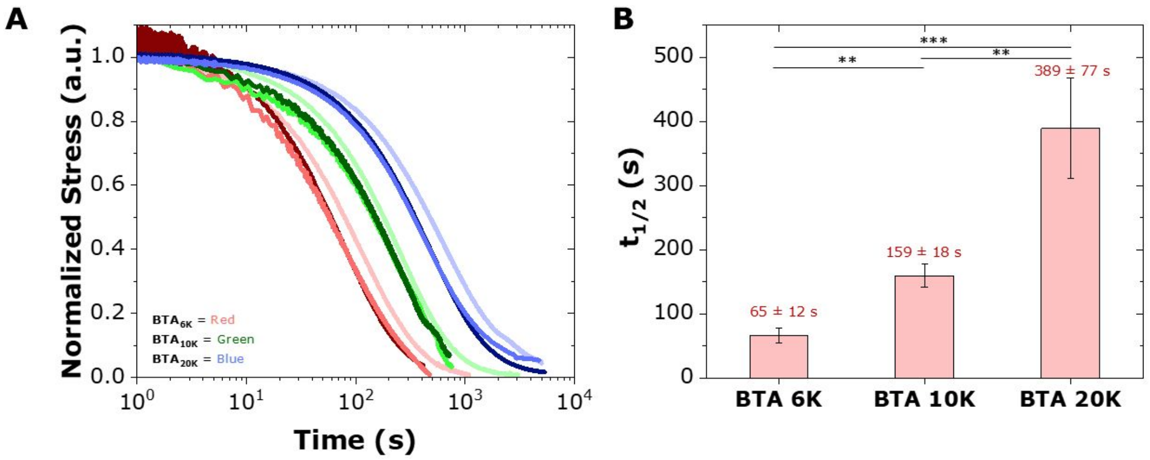

Figure S26: Repeat stress relaxation ($t_{1/2}$) measurements. A) Stress relaxation curves for BTA_{6K}, BTA_{10K}, and BTA_{20K} measured on an HR20 (TA Instruments) (dark shades) were overlaid with the corresponding data from Figure 3D measured on an MCR702 rheometer (Anton Paar, light shades). B) Relaxation half-times $t_{1/2}$ shown as mean ± SD (n = 3). Consistency between repeated measurements confirms reproducible stress relaxation behavior across independent batches and instruments. Differences in $t_{1/2}$ based on linker length were significant (** $p < 0.01$, *** $p < 0.001$).

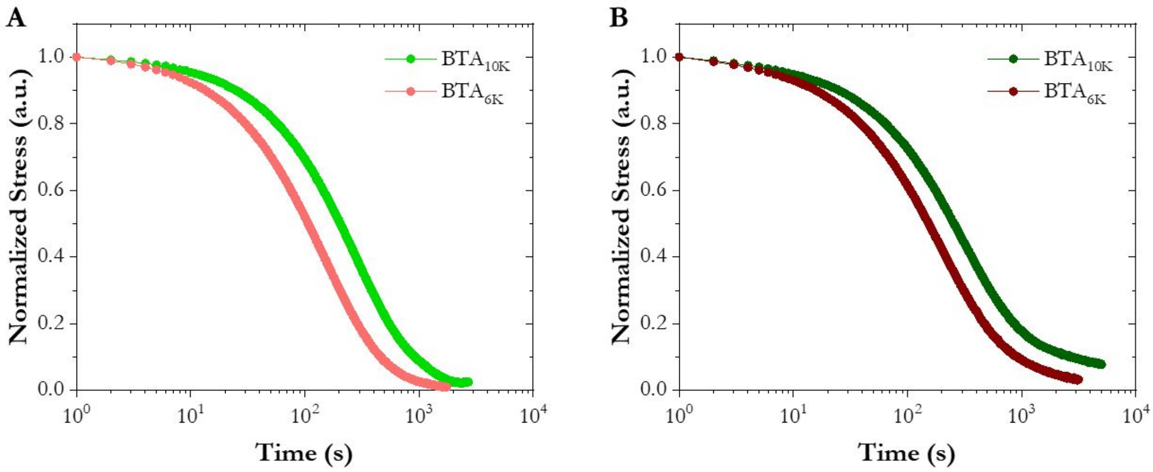

Figure S27: Stress relaxation profile of BTA_{10K} (green) and BTA_{6K} (red) at A) 15 wt% and B) 20 wt%.

Table S3: Average stress relaxation times ($t_{1/2}$) for BTA formulations to dissipate 50% of the applied stress

	$t_{1/2}$		
	**BTA_{6K}**	**BTA_{10K}**	**BTA_{20K}**
**10 wt %**	80 s	180 s	480 s
**15 wt %**	110 s	205 s	N.A.
**20 wt %**	155 s	255 s	N.A.

Empirical Fitting

$$\frac{G(t)}{G_0} = A^{\left(\frac{-t}{\tau_1}\right)} + (1-A)^{\left(\frac{-t}{\tau_2}\right)}$$

Equation S1: Maxwell model for two elements. A corresponds to the amplitude of the fast mode, τ_1 and τ_2 correspond to the fast and slow relaxation time modes, respectively.

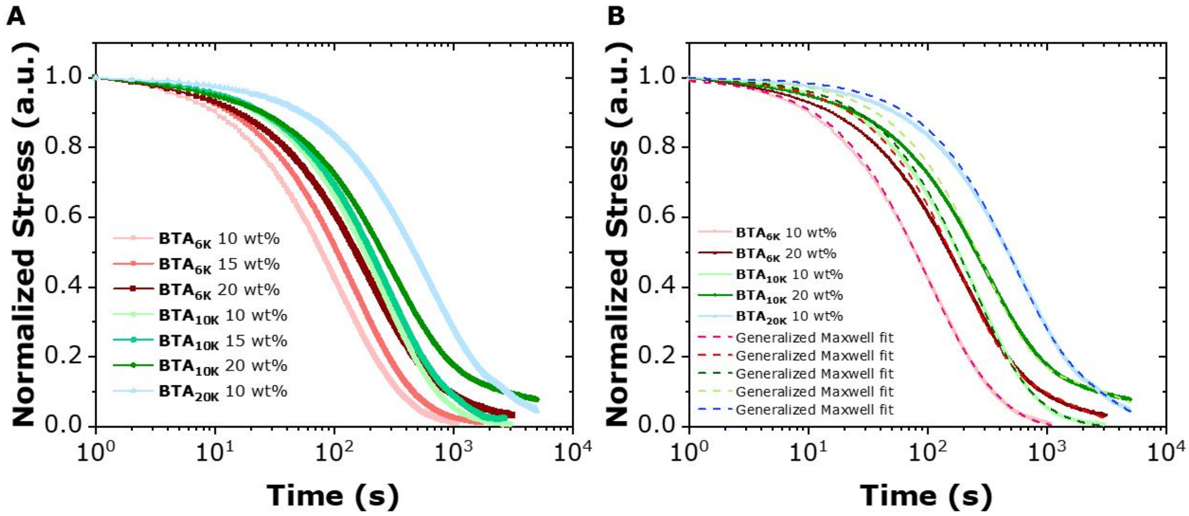

Figure S28: A) Normalized stress relaxation plots at 1% strain for all BTA hydrogels measured at 25°C. B) generalized two-element Maxwell model fit. 15 wt% hydrogels were omitted for visual clarity. R^2-values for all fits >0.997.

Table S4: $t_{1/2}$ stress relaxation timescales obtained from the normalized stress relaxation data

	**$t_{1/2}$ (generalized two-element Maxwell model)**		
	**BTA_{6K}**	**BTA_{10K}**	**BTA_{20K}**
**10 wt %**	78 s	181 s	471 s
**15 wt %**	107 s	203 s	/
**20 wt %**	156 s	261 s	/

Table S5: Values obtained for A, τ_1, and τ_2 based on the two-element Maxwell model at 25 °C. Here, τ_1 and τ_2 values are associated with the response to the applied stress of the networks' fast- and slow-relaxing elements, respectively.

	**A**		
	**BTA_{6K}**	**BTA_{10K}**	**BTA_{20K}**
**10 wt %**	0.58	0.77	0.72
**15 wt %**	0.78	0.76	/
**20 wt %**	0.82	0.83	/

	**τ_1**		
	**BTA_{6K}**	**BTA_{10K}**	**BTA_{20K}**
**10 wt %**	72	209	476
**15 wt %**	122	226	/
**20 wt %**	176	289	/

	**τ_2**		
	**BTA_{6K}**	**BTA_{10K}**	**BTA_{20K}**
**10 wt %**	244	682	2704
**15 wt %**	467	922	/
**20 wt %**	1519	5471	/

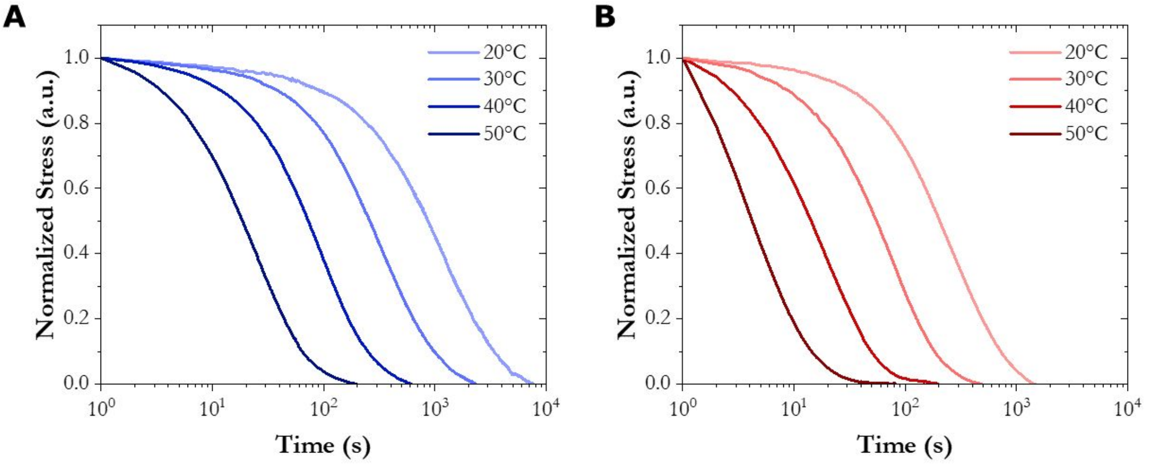

Figure S29: Temperature-dependent stress relaxation for BTA_{20K} (A) and BTA_{6K} (B) hydrogels at 1% strain.

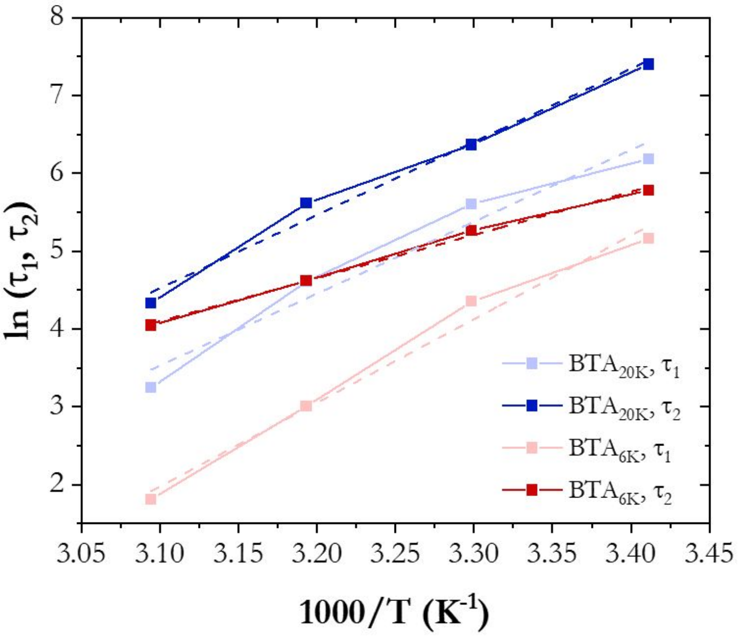

Figure S30: Arrhenius plot of τ_1 & τ_2 of BTA_{6K} and BTA_{20K} hydrogels at 20, 30, 40, and 50°C. A linear fit (dashed lines) was used to obtain the slope for every τ-value. The fit parameters are listed in Table S6.

$$ln\, \tau = mx + b, \qquad x = \frac{1000}{T}$$

Equation S2: Temperature-dependent relaxation times were fitted to the linearized Arrhenius form, where the slope *m*, intercept *b*, and coefficient of determination R^2 were obtained from linear regression.

Table S6: Temperature-dependent Arrhenius Fit parameters (intercept, slope, R^2) for the two relaxation modes of each sample obtained from Equation 2.

Sample	Mode	Intercept (*b*)	Slope (*m*)	R^2
BTA_{6K}	τ_1	-31.4 ± 3	10.75 ± 0.93	0.985
	τ_2	-13.10 ± 0.78	5.55 ± 0.24	0.996
BTA_{20K}	τ_1	-25.1 ± 4.4	9.25 ± 1.37	0.958
	τ_2	-24.7 ± 2.5	9.41 ± 0.78	0.986

Small Angle X-ray Scattering

$$d = \frac{2\pi}{q}$$

Equation S3: relationship between the real-space distance (d) to scattering vector (q)

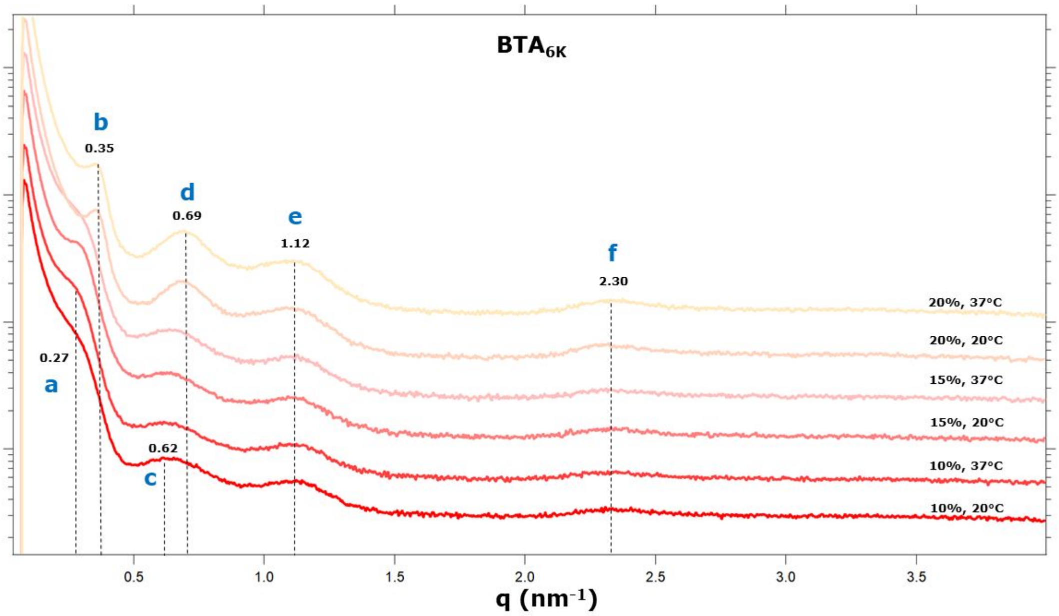

Figure S31: SAXS spectra from BTA_{6K} hydrogels at various wt % at 20°C and 37°C. Dashed lines indicate peak positions for: a, b) network mesh size; c, d, e) local BTA packing into stacks, f) single BTA units.

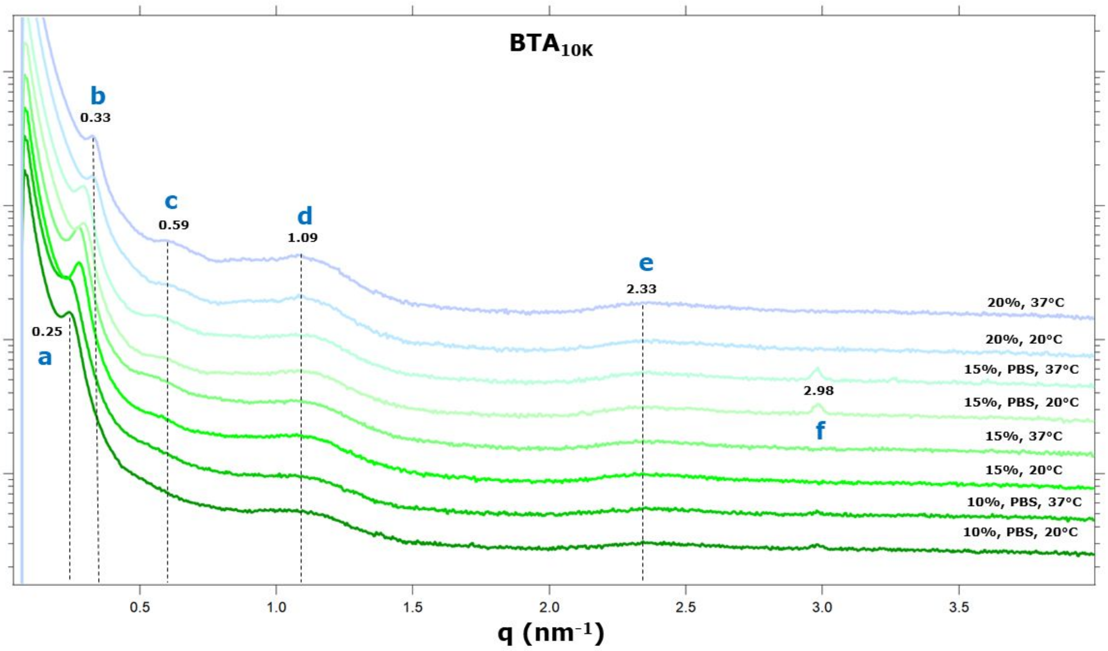

Figure S32: SAXS spectra from BTA_{10K} hydrogels at various wt % at 20°C and 37°C. Dashed lines indicate peak positions for: a, b) network mesh size; c, d) local BTA packing into stacks, e) single BTA units. No variation in q-peaks was seen for samples prepared in PBS. f) Peak position q ≈ 2.98 nm^{-1} is likely associated with PBS salts.

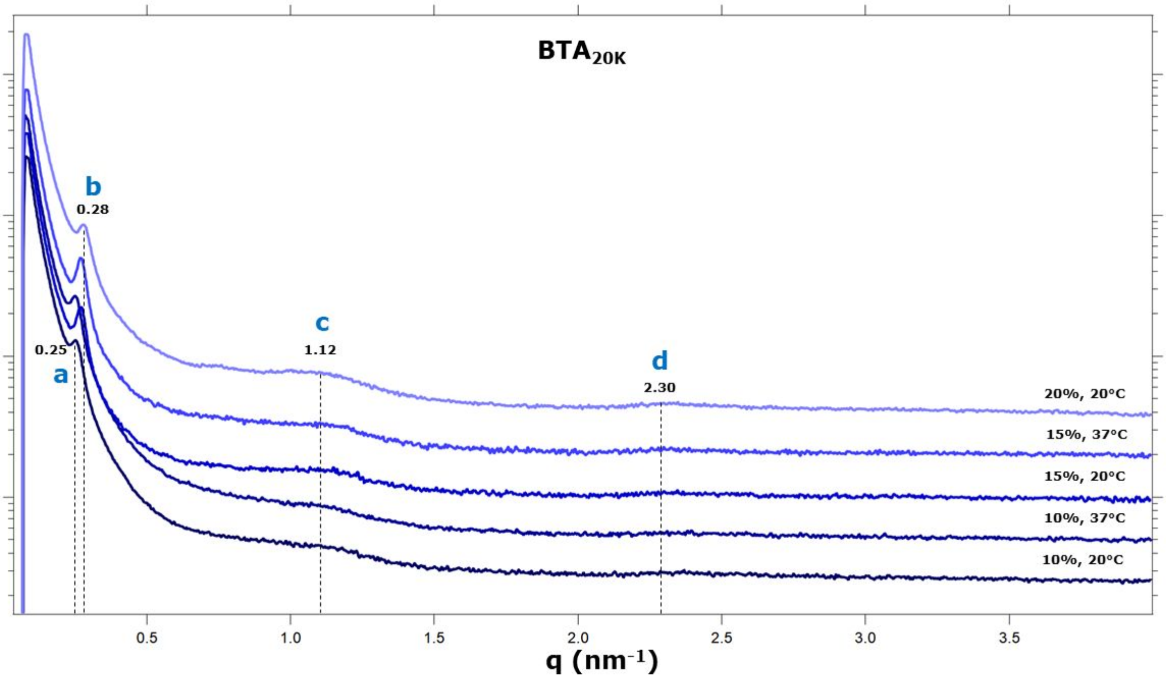

Figure S33: SAXS spectra from BTA_{20K} hydrogels at various wt % at 20°C and 37°C. Dashed lines indicate peak positions for: a, b) network mesh size; c) local BTA packing into stacks, d) single BTA units.

BTA Blend rheology

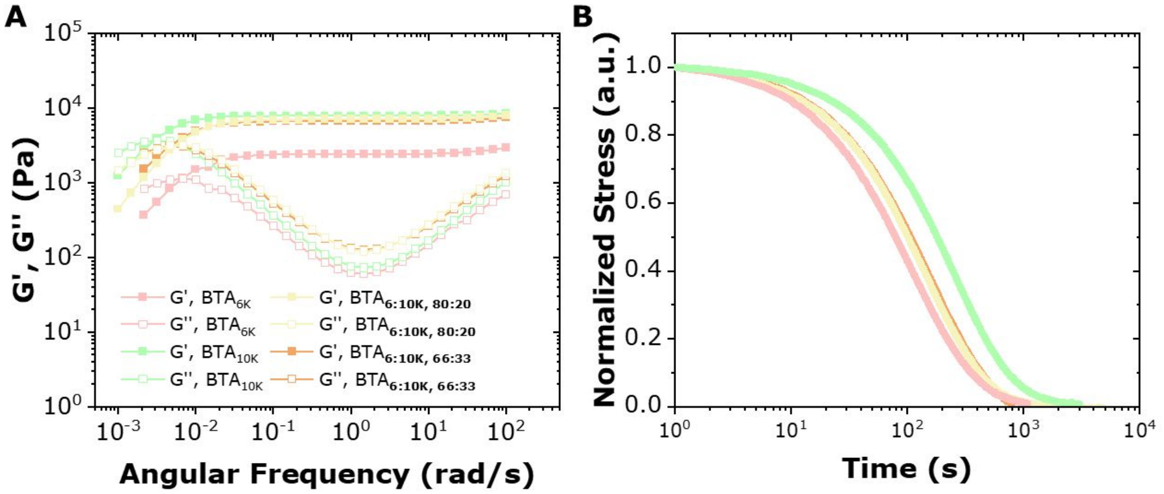

Figure S34: A) frequency sweep of BTA_{6K} and BTA_{10K}, alongside their respective 80:20 and 66:33 blends (at 10 wt %). G' increases more strongly with higher BTA_{10K} content. B) Normalized stress relaxation profile for all four formulations at 1% strain. $t_{1/2}$ shows a more modest increase with increasing BTA_{10K} content, less pronounced than the change in G'.

Table S7: Stiffness (G') of BTA blends (10 wt %).

	$BTA_{6:20K}$	$BTA_{6:10K}$
**80:20**	5.6 kPa	6.6 kPa
**66:33**	11.8 kPa	7.0 kPa

Table S8: Experimental $t_{1/2}$ stress relaxation timescales obtained from the normalized stress relaxation data

	$t_{1/2}$	
	$BTA_{6:20K}$	$BTA_{6:10K}$
**80:20**	127 s	100 s
**66:33**	181 s	106 s

Extrusion Based 3D Printing

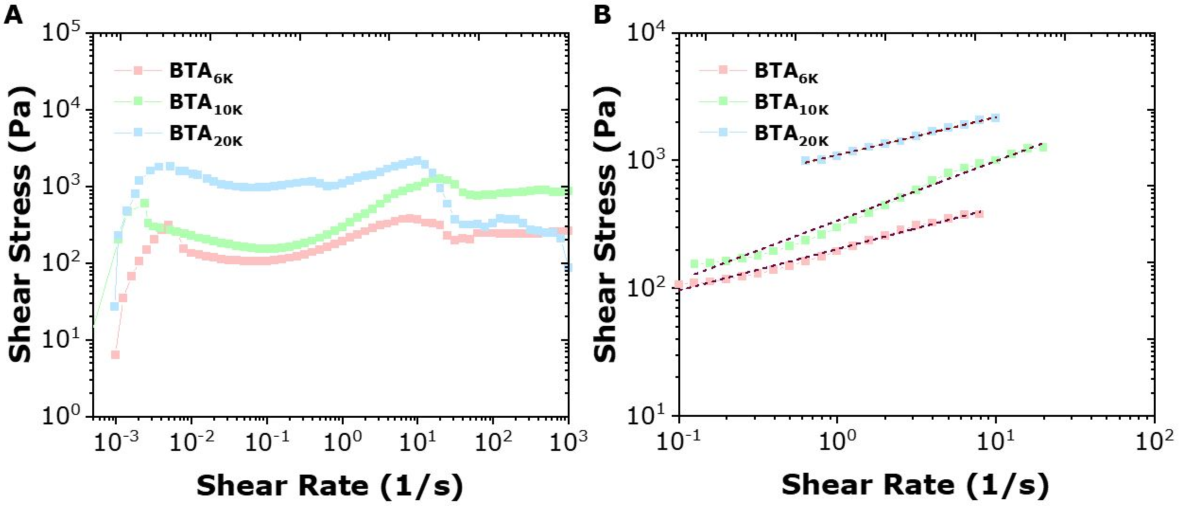

Figure S35: Shear stress vs shear rate plots of $BTA_{6K,\ 10K,\ 20K}$ hydrogelators at 10 wt %. A) Full shear-stress response across shear rates 10^{-3} to 10^{3} s^{-1}. All samples exhibit non-Newtonian behavior with a pronounced shear thinning regime through shear-induced network rearrangements at high shear rates (>10 s^{-1}). For $BTA_{20K,}$ the slight decrease in measured shear stress at shear rates >10 s^{-1} can be attributed to wall slip effects between the sample-geometry interface. B) Expanded view of the shear thinning region selected for empirical fitting of all BTA hydrogelators with the power law equation $\tau = K\dot{y}^{n}$ (dark dashed lines, extracting the consistency index K and the flow index n.

Table S9: Obtained values for K and n after fitting the data with the power law model $\tau = K\dot{y}^{n}$ with K = consistency index; n = flow index, $\dot{y}$ = shear rate (s^{-1})

	**K** (Pa s^n)	**n**
**BTA_{6K}**	202.7 ± 2.7	0.33 ± 0.01
**BTA_{10K}**	338 ± 11	0.47 ± 0.01
**BTA_{20K}**	1105 ± 11	0.30 ± 0.01

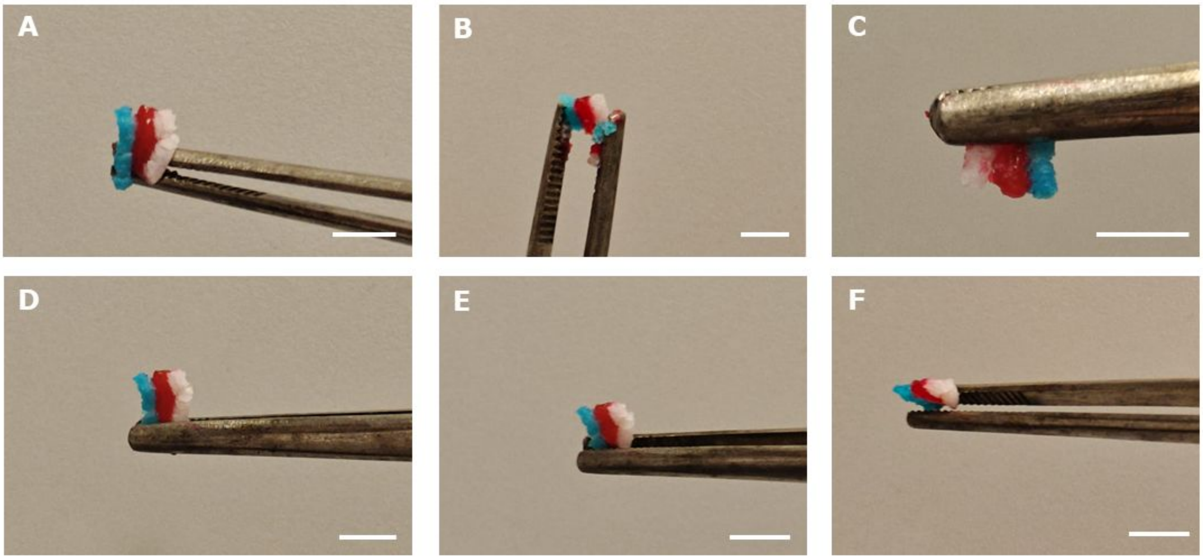

Figure S36: A-F) The 3-component hydrogel construct remains fully self-supporting while sustaining structural cohesion during repeated handling and manipulation with tweezers. Scale bar = 8 mm
